# Comparison of photosynthetic responses between haptophyte *Phaeocystis globosa* and diatom *Skeletonema costatum* under phosphorus limitation

**DOI:** 10.3389/fmicb.2023.1085176

**Published:** 2023-01-23

**Authors:** Xiaojie Chai, Lingling Zheng, Jin Liu, Jiao Zhan, Lirong Song

**Affiliations:** ^1^State Key Laboratory of Freshwater Ecology and Biotechnology, Key Laboratory of Algal Biology, Institute of Hydrobiology, Chinese Academy of Sciences, Wuhan, China; ^2^College of Life Science, University of Chinese Academy of Sciences, Beijing, China

**Keywords:** *Skeletonema costatum*, *Phaeocystis globosa*, succession, phosphorus limitation, photosynthetic response

## Abstract

The diatom *Skeletonema costatum* and the haptophyte *Phaeocystis globosa* often form blooms in the coastal waters of the South China Sea. *Skeletonemacostatum* commonly dominates in nutrient enrichment coastal waters, whereas *P*. *globosa* starts flourishing after the diatom blooms when phosphorus (P) is limited. Therefore, P limitation was proposed to be a critical factor affecting diatom–haptophyte transition. To elucidate the tolerance to P limitation in *P*. *globosa* compared with *S*. *costatum*, the effect of P limitation on their photosystem II (PSII) performance was investigated and their photosynthesis acclimation strategies in response to P limitation were evaluated. P limitation did not affect the growth of *P*. *globosa* over 7 days but decreased it for *S*. *costatum*. Correspondingly, the PSII activity of *S*. *costatum* was significantly inhibited by P limitation. The decline in PSII activity in *S*. *costatum* under P limitation was associated with the impairment of the oxygen-evolving complex (the donor side of PSII), the hindrance of electron transport from Q_A_^−^ to Q_B_ (the acceptor side of PSII), and the inhibition of electron transport to photosystem I (PSI). The 100% decrease in D1 protein level of *S*. *costatum* after P limitation for 6 days and PsbO protein level after 2 days of P limitation were attributed to its enhanced photoinhibition. In contrast, *P*. *globosa* maintained its photosynthetic activity with minor impairment of the function of PSII. With accelerated PSII repair and highly increased non-photochemical quenching (NPQ), *P*. *globosa* can avoid serious PSII damage under P limitation. On the contrary, *S*. *costatum* decreased its D1 restoration under P limitation, and the maximum NPQ value in *S*. *costatum* was only one-sixth of that in *P*. *globosa*. The present work provides extensive evidence that a close interaction exists between the tolerance to P limitation and photosynthetic responses of *S*. *costatum* and *P*. *globosa*.

## 1. Introduction

The diatom *Skeletonema costatum* and the haptophyte *Phaeocystis globosa* are often detected in the coastal water of the South China Sea (SCS) and found to form intensive blooms frequently (Liu and Tang, 2012; Wang X. D. et al., 2021; Wang et al., 2022). *Skeletonema* *costatum* usually dominates in nutrient enrichment coastal waters and is the most frequently occurring bloom species in some areas of the SCS such as the eastern Guangdong coastal region (Li et al., 2019) and Xiamen Bay (Chen et al., 2021) over the past 30 years. Although *S*. *costatum* is nontoxic, its blooms generate huge biomass influencing not only biogeochemical cycling but also the food chain (Falkowski et al., 1998; Armbrust, 2009). *Phaeocystis* *globosa* is a haptophyte species with ecological significance and can form harmful algal blooms that cause gill damage and hypoxia of fish, and its production of hemolytic toxins can contaminate seafood or kill fish. *P*. *globosa* has caused blooms almost every year in Chinese coastal waters since 1997 when the first occurrence was recorded (Chen et al., 1999). Wang K. et al. (2021) estimated that 80.6% of the *P*. *globosa* blooms in China occur in the SCS. *P*. *globosa* blooms in SCS usually occur after diatom blooms in the period from November to March (Chen et al., 2009; Wang K. et al., 2021). This phytoplankton succession in spring from diatoms to *P*. *globosa* is also a common pattern observed in the North Sea coast (Peperzak et al., 1998; Cadee and Hegeman, 2002), the Belgian coastal zone (Lancelot et al., 2005), and the Wadden Sea (Ly et al., 2014). *S*. *costatum* is also one of the common early succession species of the diatom–*P*. *globosa* (Wang X. D. et al., 2021). The latest *S*. *costatum*–*P*. *globosa* succession in SCS was recorded in Mirs Bay in late January 2021 (Liu et al., 2021).

The succession of dominant species in water bodies is considered to be regulated by numerous environmental factors and the interplay of multiple factors (Deng et al., 2014; Lu et al., 2022). The “silicate-*Phaeocystis* hypothesis” has historically been a major explanation for the appearance of *Phaeocystis* because environmental silicate concentration may determine the duration and stability of the diatom community (Lancelot et al., 1987; Reid et al., 1990). Moreover, some authors have considered the effects of temperature (Jahnke, 1989), iron availability (Schoemann et al., 2005), irradiance (Escaravage et al., 1995), and the capacity to escape grazing (Peperzak et al., 1998) on the succession from diatoms to *P*. *globosa*. Phosphorus (P) limitation often occurs in various parts of the oceans (Thingstad et al., 2005; Lin et al., 2016) as P is rapidly consumed for photosynthesis in the euphotic zone and its resupply is slow (White and Dyhrman, 2013). P limitation may also be a critical factor affecting the diatom–*P*. *globosa* transition (Lin et al., 2016; Karasiewicz et al., 2018). *Skeletonema*. *costatum* often dominates in nutrient-rich water because of its rapid growth rate related to its higher photosynthetic rate per unit carbon (Chan, 1980; Armbrust, 2009). The growth of *S*. *costatum* collapsed soon after P limitation (Ou et al., 2008; Huang et al., 2020). Ecologically, diatoms and *P*. *globosa* usually coexist during the initial phase of the spring bloom (Wang et al., 2013), but *P*. *globosa* rapidly increases at the start of the late bloom although phosphate concentrations are low (Burson et al., 2016; Liu et al., 2021). P reduction negatively affects diatoms without negative effects on *Phaeocystis* (Gypens et al., 2007).

*Phaeocystis globosa* appears to be more tolerant to P limitation than *S*. *costatum*. One of the most important mechanisms for coping with P limitation is the utilization of dissolved organic phosphorus (DOP). Although *S*. *costatum* and *P*. *globosa* have not been directly compared, it is clear that both species can exploit DOP when orthophosphate is low (Ghyoot et al., 2015; Ou et al., 2015). *S*. *costatum* has a much lower affinity for DOP compared with the dinoflagellate *Prorocentrum donghaiense* (Ou et al., 2008), which also often succeeds after the *S*. *costatum* bloom (Lu et al., 2022). Compared with diatoms, more tolerance by *P*. *globosa* to P limitation is likely because of its lower P demand (Riegman et al., 1992). *S*. *costatum* has been thought to require more phosphate because it has a high ATP content associated with a rapid growth rate (Egge, 1998). In addition, the unusual heteromorphic life cycle of *P*. *globosa* that includes gelatinous colonies and solitary cells might also contribute to its better performance under P limitation. The colony matrix of *P*. *globosa* can act as an energy and nutrient reservoir (Veldhuis et al., 1991). The solitary cells of *P*. *globosa* can compete for limiting nutrients because of their large surface area/volume ratio and their ability to move to the deeper nutrient-rich water (Verity and Medlin, 2003; Zhuang et al., 2022). Recently, Koppelle et al. (2022) demonstrated that *P*. *globosa* can utilize a phago-mixotrophic feeding strategy to acquire P and other nutrients by ingesting bacteria, which may also contribute to its remarkable success in forming blooms under P-limited conditions.

Photosynthesis is one of the most critical processes in the primary production of phytoplankton. A close interaction exists between the tolerance to P limitation and photosynthesis. Photosynthetic activity may affect the tolerance to P limitation because the energy sources for inorganic phosphate (Pi) uptake and alkaline phosphatase (APase) synthesis, which participate in the hydrolysis of DOP into phosphate, in microalgae are mostly derived from photosynthesis (Pandey, 2006; Gorbunov and Falkowski, 2022). P limitation causes a decline in photosynthesis in several different ways, including decreasing CO_2_ assimilation by reducing the regeneration of ribulose-1,5-bisphosphate in the Calvin cycle (Lewis et al., 1994; Campbell and Sage, 2006) as well as inhibiting electron transport between photosystem II (PSII) and photosystem I (PSI; Van Rensen and Vredenberg, 2009; Carstensen et al., 2018). P limitation selectively affected the PSII machinery in the model green alga *Chlamydomonas reinhardtii* with retardation of electron flow from Q_A_^−^ to Q_B_ (with Q_A_ and Q_B_ being the first and second quinone electron acceptors of PSII, respectively; Wykoff et al., 1998) and a drop in the level of the reaction center protein D1 (Allen et al., 2007). In addition, some studies have demonstrated that the regulation of various strategies for photosynthesis acclimation affects the tolerance to P limitation of phytoplankton. Enhancement of non-photochemical quenching (NPQ) helped avoid photodamage from excess light energy for coping with P deprivation in dinoflagellate *Karlodinium veneficum* (Cui et al., 2017). However, decreased PSII repair capacity in *Dunaliella tertiolecta* increased its susceptibility to photoinhibition under P limitation (Heraud et al., 2005). Although the photosynthetic activity of *S*. *costatum* (Liu et al., 2013), *P*. *donghaiense* (Shi et al., 2017), and some other species (Li et al., 2021) are significantly inhibited by P limitation, the mechanisms for the decline in photosynthetic activity are poorly studied in marine species with ecological significance, and the photosynthetic response of *P*. *globosa* under P limitation remains to be explored.

This study hypothesized that *P*. *globosa* has a higher capacity to sustain its photosynthetic activity under P limitation compared with diatom *S*. *costatum*, which contributes to their different tolerance to P limitation and affects their survival, dominance, and succession.

## 2. Materials and methods

### 2.1. Culture conditions and experimental design

Experimental strains *S*. *costatum* and *P*. *globosa* were both isolated from the coastal water in the SCS and cultured in Erdschreiber’s medium (modified from the original Plymouth seawater recipe, Rosowski and Parker, 1971; Supplementary Table S1) at (24 ± 1)°C and 50 μmol photons m^−2^ s^−1^ under a 24 h light: 0 h dark cycle. *Phaeocystis*. *globosa* was kept in the solitary form throughout the experimental period, and colony formation was not observed. This could be explained by no environmental stresses, such as grazing, in the laboratory. Na_2_SiO_3_·9H_2_O with a final concentration of 106 μM was added to the culture of *S*. *costatum*. Erdschreiber’s medium lacking Na_2_HPO_4_ was used as a P-free medium in which Pi-containing compounds were substituted with NaCl. Algal cells in the logarithmic growth phase under P-repleted condition were collected by centrifugation, washed three times with P-free Erdschreiber’s medium, and resuspended into 500 mL Erlenmeyer flasks containing 250 mL Erdschreiber’s medium (PO_4_^3−^ concentration: 67.0 μM) as the control group (+P) and 250 mL P-free medium (PO_4_^3−^ concentration: 0.0 μM) as the treatment group (−P). The initial biomass of experimental algae was set to an OD_730_ of 0.15 corresponding to Chl *a* content of ~0.6 μg/mL in *S*. *costatum* and ~ 0.4 μg/mL in *P*. *globosa*. All cultures were shaken three times every day during the 7-day cultivation period. All treatments were performed in triplicates.

To test the effect of intracellular P on the growth of *S*. *costatum* and *P*. *globosa*, the contents of polyphosphate body (PolyP) in cells were quantified according to Martin and Van Mooy (2013). Cells were harvested by centrifugation (Eppendorf 5810R centrifuge, 4,000 × g, 3 min). 10 μL proteinase K (20 mg/mL) was added to the pellets and incubated at 37°C for 30 min. Then centrifuged for 1 min at 16100 × g and the supernatant was removed. PolyP contents of samples were measured after the addition of DAPI (final concentration: 100 μM) and made on a fluorescence spectrofluorometer (F-2700, Hitachi, Japan) at an excitation wavelength of 415 nm and emission wavelength of 550 nm, with an integration time of 0.5 s.

Various chlorophyll fluorescence tools combined with protein measurements were used to examine the different effects of P limitation on PSII in *S*. *costatum* and *P*. *globosa*. The photosynthetic acclimation mechanisms of *S*. *costatum* and *P*. *globosa* were explored in terms of induction of NPQ and D1 turnover.

### 2.2. Growth and maximum photochemistry efficiency (F_v_/F_m_)

Growth characteristics of cells were studied by monitoring the optical density at 730 nm (OD_730_) using a spectrophotometer. F_v_/F_m_ was obtained from the JIP-test after dark-adapted for 15 min at room temperature according to Strasser et al. (2004) with a dual modulation kinetic fluorometer FL3500 (PSI, Brno, Czechia). These two values were obtained from cultures every day.

### 2.3. Rapid light response curve

The rapid light response curve included steps of actinic irradiance: 2, 7, 14, 28, 45, 64, 92, 131, 181, 245, 321, 412, 540, 684, 869, 1,109, and 1,410 μmol·m^−2^ s^−1^, with a 30-s interval between steps using a Dual-PAM 100 (Walz, Effeltrich, Germany) under the illumination with the measuring light at 460 nm and the red actinic light at 635 nm. The built-in fitting function was used to fit the rapid light response curves with the EP model and derive the maximum electron transport rate (ETR_max_), light-harvesting efficiency (alpha), and the point of light saturation (Ik).

### 2.4. Polyphasic Chl *a* fluorescence transient

Samples were dark-adapted for 15 min at room temperature before fluorescence measurement using a dual modulation kinetic fluorometer FL3500 (PSI, Brno, Czechia). Under conditions of continuous red actinic light (630 nm) at a high intensity of 2000 μmol·m^−2^ s^−1^, the Chl *a* fluorescence transient was recorded up to 1 s on a logarithmic timescale, with data acquisition every 10 μs for the first 2 ms and for every 1 ms thereafter. The curves were normalized between F_o_ and F_m_ and plotted as V_t_ changes on a logarithmic scale [V_t_ = (F_t_ − F_o_)/(F_m_ − F_o_)], where F_t_ is the fluorescence in respective time, F_o_ is the minimal fluorescence and F_m_ is the maximum fluorescence. The quantum yield of electron transport (φE_0_), the reduction in end acceptors on the PSI electron acceptor side (RE_0_/RC), and the amount of active PSII RCs per CS (RC/CS_0_) values were obtained from the JIP-test.

### 2.5. Measurement of Q_A_^−^ reoxidation kinetics

Samples of 3 mL were adjusted to an OD_730_ of 0.7 and dark-adapted for 10 min before measurement. The Q_A_^−^ reoxidation kinetics after a single turnover flash was performed with a double-modulation fluorometer FL6600 (Photon Systems Instruments, Brno, Czechia; Eisenstadt et al., 2008). Both measuring flashes (4 μs) and actinic flashes (50 μs) were provided by computer-controlled light-emitting diodes. Q_A_^−^ reoxidation kinetic curves were normalized and plotted as [F_(t)_-*F*_(0)_] changes on a logarithmic scale, where F_(t)_ is the fluorescence in respective time and F_(0)_ is the initial fluorescence.

The relaxation of the flash-induced increase in Chl *a* fluorescence yield reflects the reoxidation of Q_A_^−^ *via* forward electron transport to Q_B_ and reverse reactions with the S_2_ state of the oxygen-evolving complex (OEC; Cao and Govindjee, 1990; Dau, 1994). The fast phase is attributable to the reoxidation of Q_A_^−^ by Q_B._ The middle phase arises from Q_A_^−^ reoxidation in the PSII reaction center that has an empty Q_B_ site at the time of the flash and has to bind to a plastoquinone (PQ) molecule from the PQ pool. The slow phase reflects Q_A_^−^ reoxidation with the S_2_ state of the OEC, thus causing backward electron transport through the equilibrium of Q_A_^−^Q_B_ and Q_A_Q_B_^−^ (Zhang et al., 2017). When Q_A_^−^ reoxidation kinetics were determined in the presence of 20 μM 3-(3,4-dichlorophenyl)-1,1-dimethylurea (DCMU), the fluorescence decay reflects Q_A_^−^ reoxidation *via* charge recombination with the S_2_ state of the OEC (Vass et al., 1999).

According to Beauchemkin et al. (2007), the Q_A_^−^ reoxidation kinetics curves were fitted by the three-exponential component decay equation:

F_(t)_ = A_1_ exp. (−t/T_1_) + A_2_ exp. (−t/T_2_) + A_3_ exp. (−t/T_3_) + A_0_

Where F(t) is the variable fluorescence yield, A_0_ to A_3_ are the amplitudes, and T_1_ to T_3_ are the time constants from which the half-life values can be calculated as t_1/2_ = ln 2 T.

### 2.6. S-state test

To further determine the proportion of inactive PSII centers (PSII_X_), S-state tests were performed with FL6600 after concentrated and dark-adapted samples as described in section 2.5. The population of the PSII_X_ center was measured by the difference between F_o_ and the fluorescence level 100 ms after the fourth flash (F_4_ = F_400ms_/F_o_ − 1) because the fluorescence decay after the fourth flash is controlled almost entirely by inactive centers (Lavergne and Leci, 1993). Given the decreased relative variable fluorescence, a revised equation was proposed according to Pan et al. (2008) as follows: PSII_X_(%) = F_4_ × 100/(F_300ms_/F_o_ − 1).

### 2.7. Measurement of non-photochemical quenching

Samples were dark-adapted for 30 min before measurement using a Dual-PAM 100. The slow induction kinetic curves at a specific process (Delay: 30 s, Measure light: 46 μmol photons m^−2^ s^−1^, Saturable Pulse: 20000 μmol photons m^−2^ s^−1^, Width: 300 ms, Clock: 1 min). NPQ was calculated as Bilger and Bjorkman (1990): NPQ = (Fm − Fm′)/Fm′, where Fm′ is the maximum fluorescence measured after the samples are exposed to continuous red actinic light (635 nm) of 321 μmol photons m^−2^ s^−1^.

### 2.8. Total cellular protein extraction, SDS-PAGE analysis, and immunoblotting

Total cellular proteins were extracted as previously described by Thangaraj et al. (2021) with minor modifications. Cells were harvested and resuspended in lysis buffer (40 mM Tris–HCl, pH 8.0) supplemented with 1 mM phenylmethanesulfonyl fluoride (PMSF) as the protease inhibitor. The cells were lysed using a noise isolating chamber (3 s on, 3 s off, Scientz, China) for 20 min on ice with a whole-cell lysate and then centrifuged (Eppendorf 5810R centrifuge) at 1,800 × g for 10 min at 4°C to remove cell debris. Protein concentrations were determined using the BCA assay (Beyotime, China).

Protein samples were subjected to 12.5% SDS-PAGE where each gel lane was loaded with equal amounts of 10 μg, stained with Coomassie brilliant blue R250, or transferred to polyvinylidene fluoride membranes. Subsequently, each membrane was blocked for 1 h in 5% skimmed milk and probed using rabbit primary anti-D1 (1:4000, PhytoAB, United States) and anti-PsbO (1:2000, PhytoAB, United States) antibodies. Immunodetection was performed using a goat-anti-rabbit secondary antibody conjugated to horseradish peroxidase (1:5000). Proteins were visualized on the basis of the intensities of immunoreactions using an ImageQuant LAS 4000 Mini system (GE Healthcare) and semi-quantitated by ImageJ 1.52a (Wayne Rasband, United States).

The clearance of the D1 protein untreated and treated with lincomycin (block counteracting repair processes, Wu et al., 2012) was plotted and compared. Samples in triplicates were supplemented with 1,000 μg/mL lincomycin and incubated in the dark for 10 min, to allow the antibiotic to penetrate the cells and inhibit ribosome function. All samples were then shifted to 50 μmol photons m^−2^ s^−1^ and collected on days 0, 1 and 4 for later protein immunodetection. Newly synthesized D1 protein in PSII repair was calculated as D1_newly_ = D1_active_ − D1_blocked_, where D1_active_ is the change in the active D1 level in the absence of lincomycin (PSII repair active), and D1_blocked_ is the change in the active D1 level in the presence of lincomycin (PSII repair blocked).

### 2.9. Statistical analyses

All experiments were performed in triplicates, and the results are presented as means ± standard deviations (SD). Statistical significance of differences between treatments was compared using two-way ANOVA with the least-significant difference (LSD) by SPSS 18.0 (IBM, United States).

## 3. Results

### 3.1. The growth characteristics and contents of PolyP of *Skeletonema costatum* and *Phaeocystis globosa*

To test the endogenous phosphorus in initial experimental cells, intracellular PolyP in *S*. *costatum* and *P*. *globosa* in +P and − P groups on days 0, 3 and 7 were measured (Supplementary Figure S1). The contents of PolyP in *S*. *costatum* and *P*. *globosa* cells were both significantly lower in the +P group compared with the −P group on day 0 (*p* < 0.01, ANOVA), indicating that the above two strains have the ability to store phosphate and form the PolyP when P is sufficient. The contents of PolyP in *S*. *costatum* and *P*. *globosa* in −P groups were 0.005 ± 0.0016 and 0.003 ± 0.0003 nmol per OD_730_ on day 0, and 0.003 ± 0.0002 and 0.002 ± 0.0008 nmol per OD_730_ on day 7, respectively (Supplementary Figure S1), which indicated that the cells in −P groups were P-limited for the entire experiment duration.

**Figure 1:**
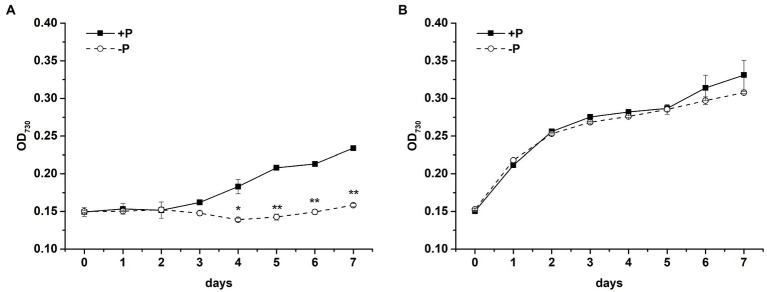
Growth curves of *S*. *costatum* **(A)** and *P*. *globosa* **(B)** in +P and −P groups. Error bars show the mean ± standard error (SE) of triplicate treatments. Superscripts indicate significant differences between +P group and −P group according to a two-way ANOVA with a *post-hoc* test (^**^p < 0.01, ^*^p < 0.05).

The cell propagation of *S*. *costatum* was completely inhibited after exposure to P limitation. The OD_730_ of cells cultured in −P group was significantly lower after 4-day cultivation (Figure 1A, *p* < 0.01, ANOVA), implying that *S*. *costatum* experienced growth inhibition under the P-limited condition. In comparison, *P*. *globosa* cell growth in the absence of P showed OD_730_ similar to that of the control (*p* > 0.05), which could still maintain a logarithmic period (Figure 1B).

### 3.2. Effects of P limitation on chlorophyll fluorescence in *Skeletonema costatum* and *Phaeocystis globosa*

#### 3.2.1. Effects of P limitation on F_v_/F_m_

**Figure 2:**
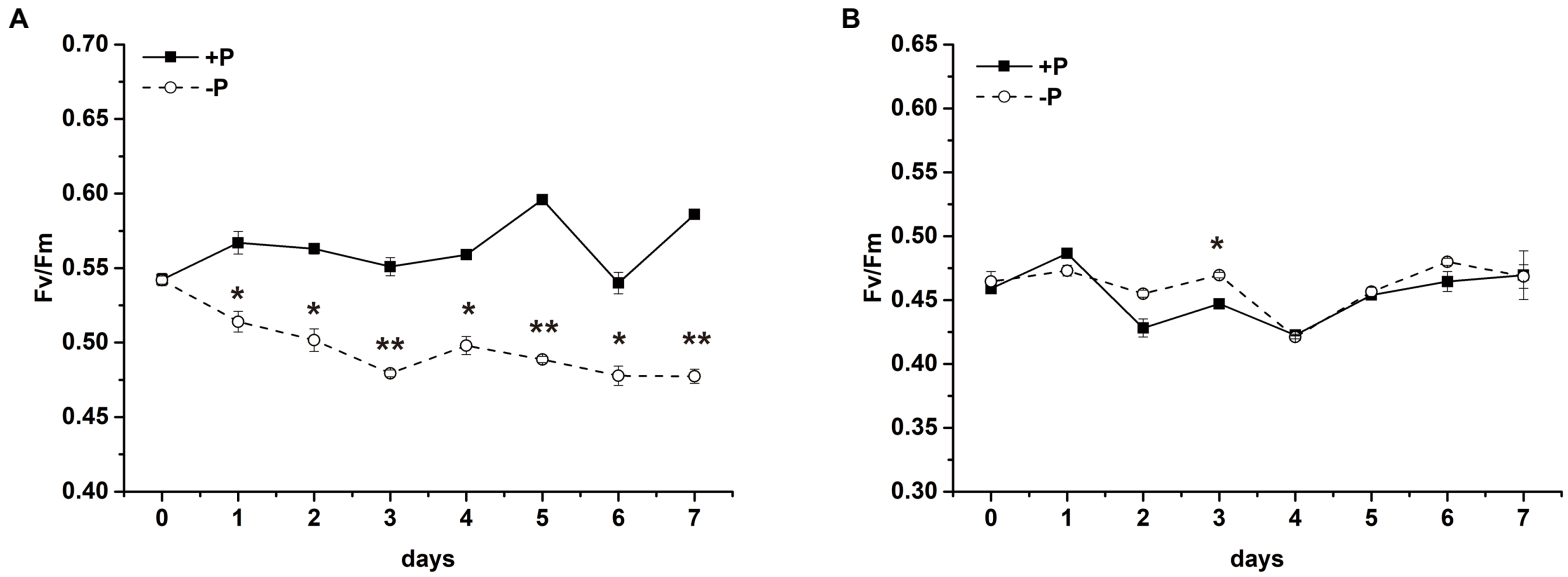
Maximum photochemistry efficiency (F_v_/F_m_) of *S*. *costatum* **(A)** and *P*. *globosa* **(B)** in +P and −P groups. Error bars show the mean ± standard error (SE) of triplicate treatments. Superscripts indicate significant differences between +P group and −P group according to a two-way ANOVA with a *post-hoc* test (^**^p < 0.01, ^*^p < 0.05).

To explore the effect of P limitation on PSII, time courses were presented for F_v_/F_m_ of PSII in *S*. *costatum* (Figure 2A) and *P*. *globosa* (Figure 2B). A difference in F_v_/F_m_ values in the +P and − P groups was observed in *S*. *costatum* (Figure 2A). F_v_/F_m_ of *S*. *costatum* decreased sharply after being cultured under P limitation (Figure 2A, *p* < 0.05, ANOVA), whereas that in the +P culture stayed at a relatively stable and higher level, indicating that P limitation led to lower photochemical efficiency in *S*. *costatum*. No significant difference in F_v_/F_m_ of *P*. *globosa* was found in the +P and − P groups (Figure 2B, *p* > 0.05, ANOVA), even after 25 days of incubation (Supplementary Figure S2B), indicating that F_v_/F_m_ of *P*. *globosa* could maintain stability under P limitation.

#### 3.2.2. Effects of P limitation on the electron transport rate and light-harvesting efficiency

**Figure 3:**
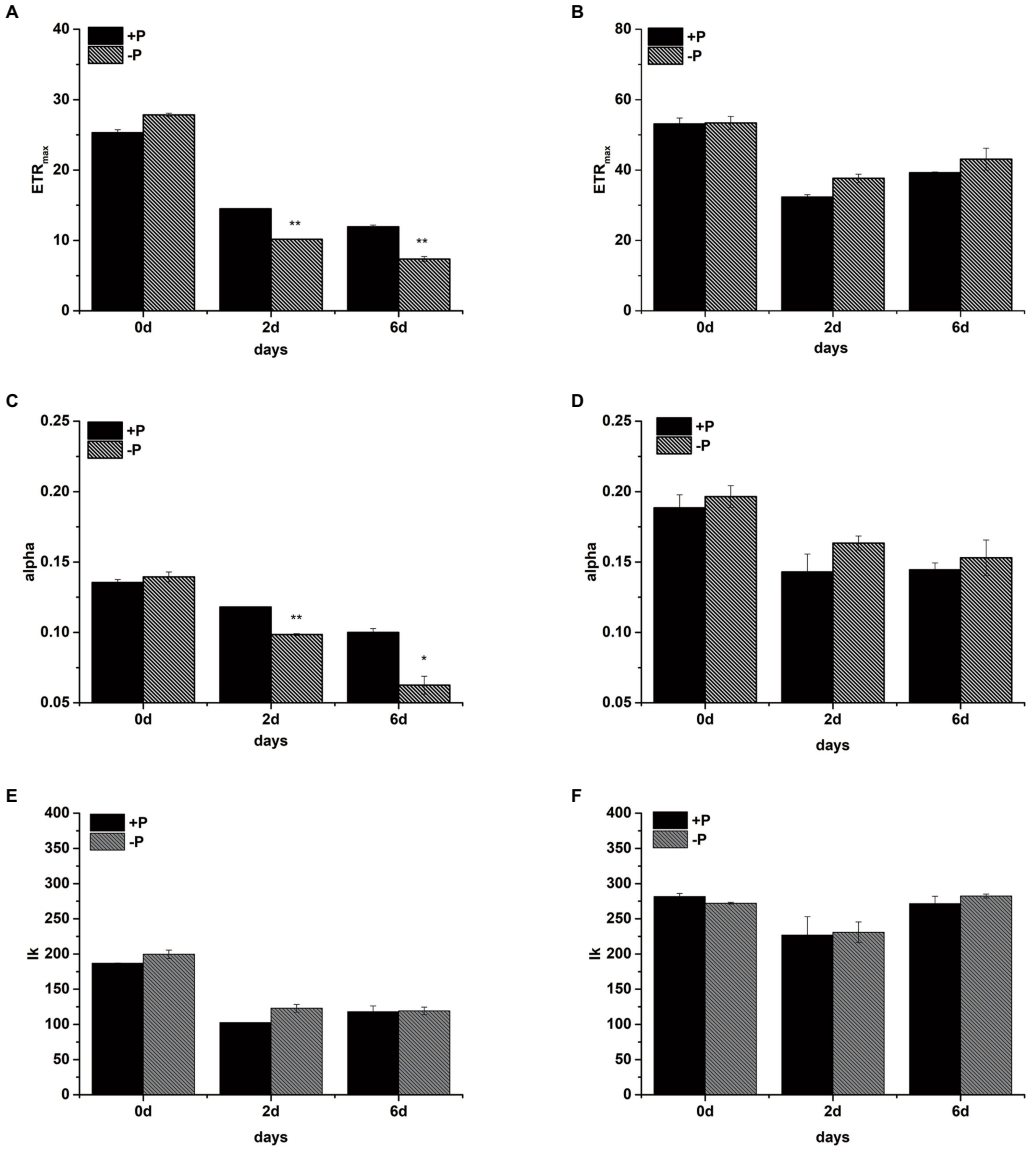
The maximum electron transport rate ETR_max_ (**A**: *S*. *costatum*, **B**: *P*. *globosa*), light-harvesting efficiency alpha (**C**: *S*. *costatum*, **D**: *P*. *globosa*), and the point of light saturation Ik (**E**: *S*. *costatum*, **F**: *P*. *globosa*) in +P and −P groups. Error bars show the mean ± standard error (SE) of triplicate treatments. Superscripts indicate significant differences between +P group and −P group according to a two-way ANOVA with a *post-hoc* test (^**^p < 0.01, ^*^p < 0.05).

The maximal electron transport rates through PSII (ETR_max_), light-harvesting efficiency (alpha), and the point of light saturation (Ik) were measured in the +P and − P groups on days 0, 2, and 6, respectively (Figure 3). After 2 days of cultivation, the ETR_max_ and alpha values of *S*. *costatum* were both significantly lower in the −P group compared with the +P group (Figures 3A,C, *p* < 0.01, ANOVA), indicating that the ability to transport the electron and to harvest the light of *S*. *costatum* cells decreased under P limitation. However, no significant decrease was observed in the above two values of the −P group compared with that of the +P group in *P*. *globosa* (Figures 3B,D). Thus, the electron transport and light-harvesting abilities of *P*. *globosa* were not limited by P limitation.

#### 3.2.3. Polyphasic Chl *a* fluorescence transient

**Figure 4:**
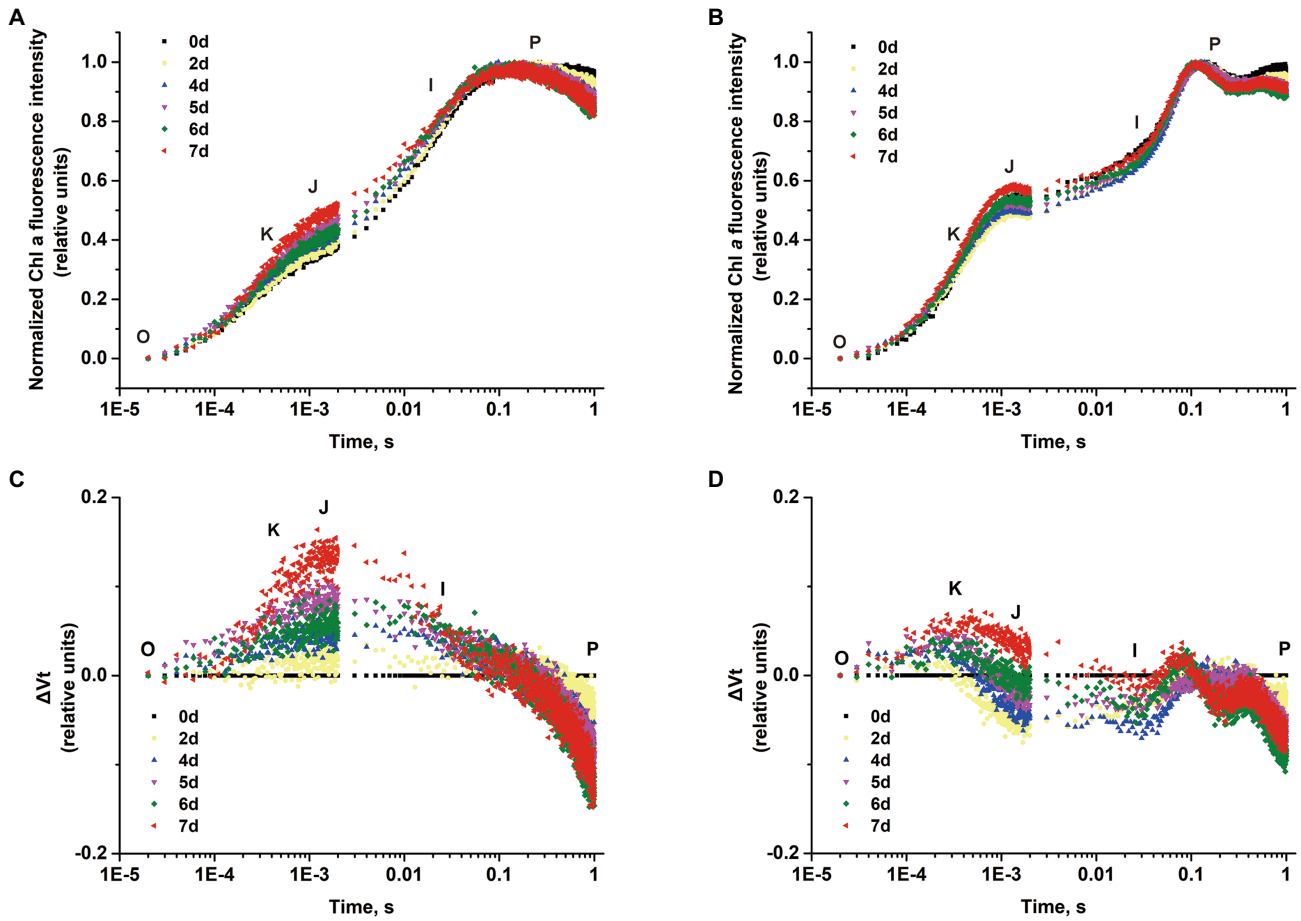
Normalized chlorophyll *a* fluorescence transient of *S*. *costatum* **(A)** and *P*. *globosa* **(B)** in −P group for various periods of time plotted by the mean of triplicate (Error bars omitted). Differential curves of ΔVt (obtained by subtracting the curve on day 0 from the samples for various periods of time) in *S*. *costatum* **(C)** and *P*. *globosa* **(D)** under P-limited condition.

The polyphasic rise of the Chl *a* fluorescence transient (OJIP) in *S*. *costatum* and *P*. *globosa* cultured with −P groups were monitored to localize the action site of P limitation in the PSII transport chain. A typical curve characteristic has four phases of O, J, I, and P (Stirbet and Govindjee, 2012), but the peak in phase I was not observed in both species in this study. The fluorescence intensity of phase J represents the accumulation of Q_A_^−^Q_B_ and Q_A_^−^Q_B_^−^ (with Q_A_ and Q_B_ being the first and second quinone electron acceptors of PSII, respectively; Zhu et al., 2005). After normalizing the curves, the variable fluorescence of phase J increased continuously in the −P group, which was found in both *S*. *costatum* (Figure 4A) and *P*. *globosa* (Figure 4B), suggesting a reduction in the rate of Q_B_-mediated Q_A_^−^ reoxidation (Haldimann et al., 1995). In addition, the increase in the K-step (300 μs) was pronounced in cells after P limitation in both *S*. *costatum* (Figure 4C) and *P*. *globosa* (Figure 4D), indicating OEC damage (Srivastava et al., 1997).

**Figure 5:**
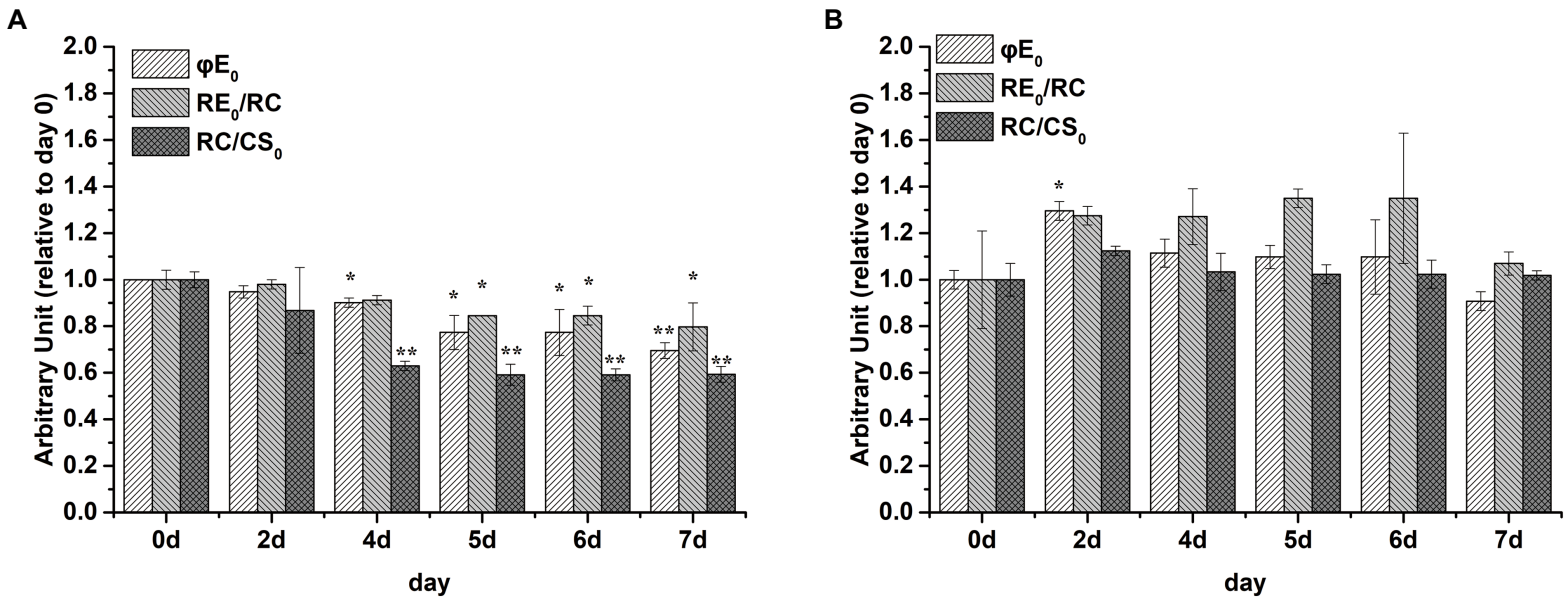
Photosynthetic parameters (relative to day 0) derived by the JIP-test of *S*. *costatum* **(A)** and *P*. *globosa* **(B)** under P-limited condition. φE_0_ = ET_0_/ABS = (F_v_/F_m_) × (1 – V_J_), which is the quantum yield of electron transport to intersystem electron acceptors at t = 0; RE_0_/RC = (RE_0_/ET_0_) – (ET_0_/RC), denoting the reduction in end acceptors on the PSI electron acceptor side per RC at t = 0; RC/CS_0_ = φP_0_ × (ABS/CS_0_) × (V_J_/M_0_), reflecting the amount of active PSII RCs per CS at t = 0. Error bars are mean ± standard error (SE) of triplicate treatments. Superscripts indicate significant differences between +P group and − P group according to a two-way ANOVA with a *post-hoc* test (^**^p < 0.01, ^*^p < 0.05).

φE_0_, RE_0_/RC, and RC/CS_0_ values were markedly decreased by P limitation compared with the corresponding values on day 0 in *S*. *costatum* (Figure 5A, *p* < 0.05, ANOVA) but not in *P*. *globosa* (Figure 5B, *p* > 0.05, ANOVA). Therefore, P limitation decreased the quantum yield of electron transport to intersystem electron acceptors and the electron flux for reducing terminal electron acceptors on the PSI side in *S*. *costatum* rather than *P*. *globosa* (Lin et al., 2009). In addition, the amount of active PSII RCs per excited CS decreased by P limitation in *S*. *costatum* (Lin et al., 2009).

#### 3.2.4. Effects of P limitation on Q_A_^−^ reoxidation kinetics

**Figure 6:**
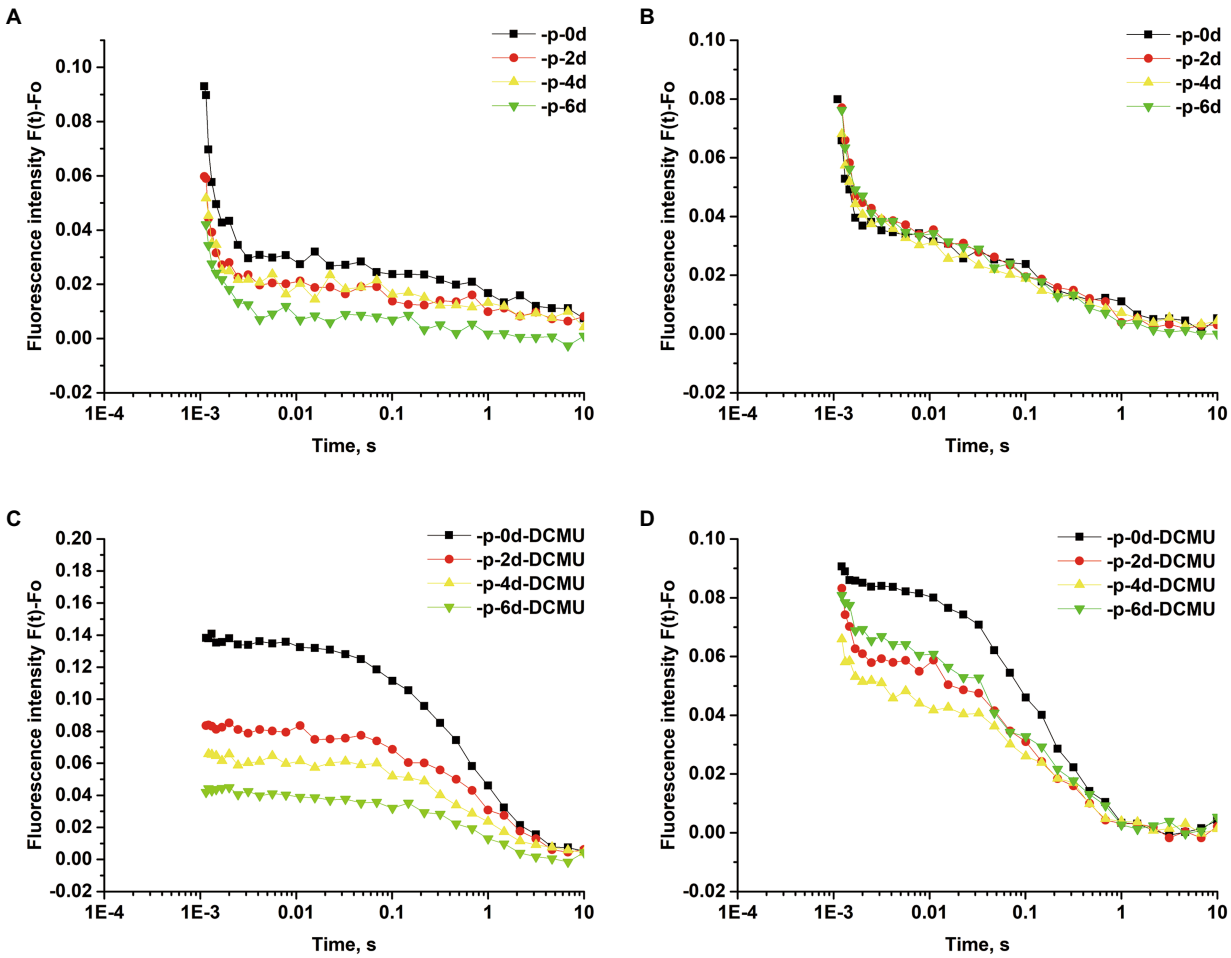
Normalized Q_A_^−^ reoxidation kinetic curves of *S*. *costatum* and *P*. *globosa* in +P and −P groups for various periods of time. Fluorescence decay in the absence of 3-(3,4-dichlorophenyl)-1,1-dimethylurea (DCMU) of *S*. *costatum* **(A)** and *P*. *globosa* **(B)**. Fluorescence decay in the presence of 20 μM DCMU of *S*. *costatum* **(C)** and *P*. *globosa* **(D)**. Each value represents the mean of the results from triplicates (Error bars omitted).

To verify the possibility of Q_A_^−^ reoxidation being retarded in response to P limitation, the Q_A_^−^ reoxidation kinetics test was performed. The curves were fitted by three-exponential decay without DCMU. The Q_A_^−^ reoxidation kinetic curves of *S*. *costatum* and *P*. *globosa* in the +P and − P groups for different periods are shown in Figure 6. The parameters of Q_A_^−^ reoxidation kinetics in cells exposed to P limitation are summarized in Table 1. P limitation increased the decay half-life of the fast phase (T1) in *S*. *costatum*, and it increased from 714.1 ± 58.4 μs on day 0 to 1010.0 ± 154.0 μs on day 6 in the −P group (Table 1, *p* < 0.05, ANOVA). For *P*. *globosa*, the decay half-life of the fast phase (T1) increased from 526.0 ± 17.0 μs on day 0 to 871.0 μs on day 6 (Table 1, *p* < 0.05, ANOVA). Therefore, P limitation retards Q_B_^−^ mediated Q_A_^−^ reoxidation in both *S*. *costatum* and *P*. *globosa* (Zhang et al., 2017), which is in accordance with the observed changes of phase J (Figure 4).

**Table 1:** Decay kinetics of flash-induced variable fluorescence in *S*. *costatum* and *P*. *globosa*.

	Time (d)	Fast phase T1 (μs)/A1 (%)	Middle phase T2 (ms)/A2 (%)	Slow phase T3 (s)/A3 (%)	A0 (%)
*Skeletonema costatum*	P without DCMU				
	0	714.1 ± 58.4^a^/40.32	0.00/0.00	2.00 ± 0.77^a^/42.07	17.61
	2	670.5 ± 151.7^a^/40.17	0.00/0.00	2.68 ± 1.09^a^/43.52	16.31
	4	822.7 ± 234.8^a^/34.31	0.00/0.00	1.57 ± 0.70^a^/41.91	23.78
	6	1010.0 ± 154.0^b^/48.08	0.00/0.00	1.26 ± 0.53^a^/47.19	4.73
	P with DCMU				
	0			0.71 ± 0.03^a^/59.10	40.9
	2			0.91 ± 0.07^b^/57.66	42.34
	4			0.72 ± 0.05^a^/55.96	44.04
	6			0.72 ± 0.08^a^/58.45	41.55
*Phaeocystis globosa*	P without DCMU				
	0	526.0 ± 17^a^/33.02	0.00/0.00	0.27 ± 0.05^a^/45.89	20.09
	2	855 ± 126^b^/34.00	0.00/0.00	0.31 ± 0.07^a^/47.76	18.24
	4	1,168 ± 168^b^/37.34	0.00/0.00	0.27 ± 0.07^a^/45.55	17.11
	6	871.0^b^/32.73	0.00/0.00	0.27 ± 0.04^a^/49.77	17.51
	-P with DCMU				
	0			0.18 ± 0.01^a^/61.44	38.56
	2			0.13 ± 0.02^b^/62.30	37.7
	4			0.14 ± 0.00^b^/61.03	38.97
	6			0.17 ± 0.02^a^/61.03	38.94

Cells were treated with P deficiency for indicated periods of time, and the relaxation of the flash-induced fluorescence yield with or without 20 μM 3-(3,4-dichlorophenyl)-1,1-dimethylurea (DCMU) was measured. The curves were analyzed in terms of three exponential components (fast, middle, and slow phases). Superscript letters indicate significant differences between day 0 and other days according to a two-way ANOVA with a *post-hoc* test (*p* < 0.05).

The curves were fitted by the single exponential decay under the presence of 20 μM DCMU. For *S*. *costatum*, there was a significant increase from 0.71 ± 0.03 μs (day 0) to 0.91 ± 0.07 μs (day 2) in the time constant for the slow phase (T3; *p* < 0.05, ANOVA), and the increase in the non-decaying component (A0) of fluorescence after P limitation (Table 1), which increased from 40.90% (day 0) to 42.34% (day 2), indicating that Q_A_^−^ was restricted to recombine with the S_2_ state after only 2 days of P-limited culture in *S*. *costatum* cells. However, no significant increase in the slow phase (T3) was observed in *P*. *globosa*, and the non-decaying component (A0) of fluorescence increased slightly from 38.56% (day 0) to 38.94% (day 6) under P limitation (Table 1).

#### 3.2.5. Effects of P limitation on the proportion of PSII_X_ centers

**Figure 7:**
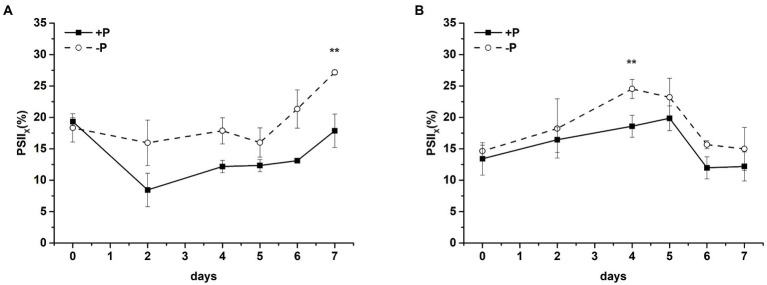
Proportion of PSII_X_ centers of *S*. *costatum* **(A)** and *P*. *globosa* **(B)** in +P and −P groups for various periods of time. Error bars show the mean ± standard error (SE) of triplicate treatments. Superscripts indicate significant differences between +P group and −P group according to a two-way ANOVA with a *post-hoc* test (^**^p < 0.01).

Intracellular PSII exists in different forms, which is the so-called heterogeneity. According to the electron transfer capacity, PSII can be divided into PSII_A_ (active PSII center) and PSII_X_ (inactive PSII center; Lavergne and Leci, 1993). In PSII_A_ centers, the oxidation of Q_A_^−^ is rapid, whereas in PSII_X_ centers, the oxidation of Q_A_^−^ is much slower (Chylla and Whitmarsh, 1989). The proportion of PSII_X_ in *S*. *costatum* and *P*. *globosa* cells treated with +P and − P for various periods of time was measured by the S-state test (Supplementary Figure S3). The proportion of PSII_X_ centers increased in *S*. *costatum* under P limitation, which significantly increased from 18.34 ± 2.26% to 27.17 ± 0.38% after 7 days of P limitation (*p* < 0.01, Figure 7A, ANOVA), indicating that the proportion of Q_B_ that cannot oxidize Q_A_^−^ increased. Similarly, P limitation had significant effects on the proportion of PSII_X_ centers in *P*. *globosa*, which increased from 14.65 ± 0.92% on day 0 to 24.54 ± 1.53% on day 4 (Figure 7B, *p* < 0.01, ANOVA).

### 3.3. Effects of P limitation on their photosystem II proteins of *Skeletonema costatum* and *Phaeocystis globosa*

**Figure 8:**
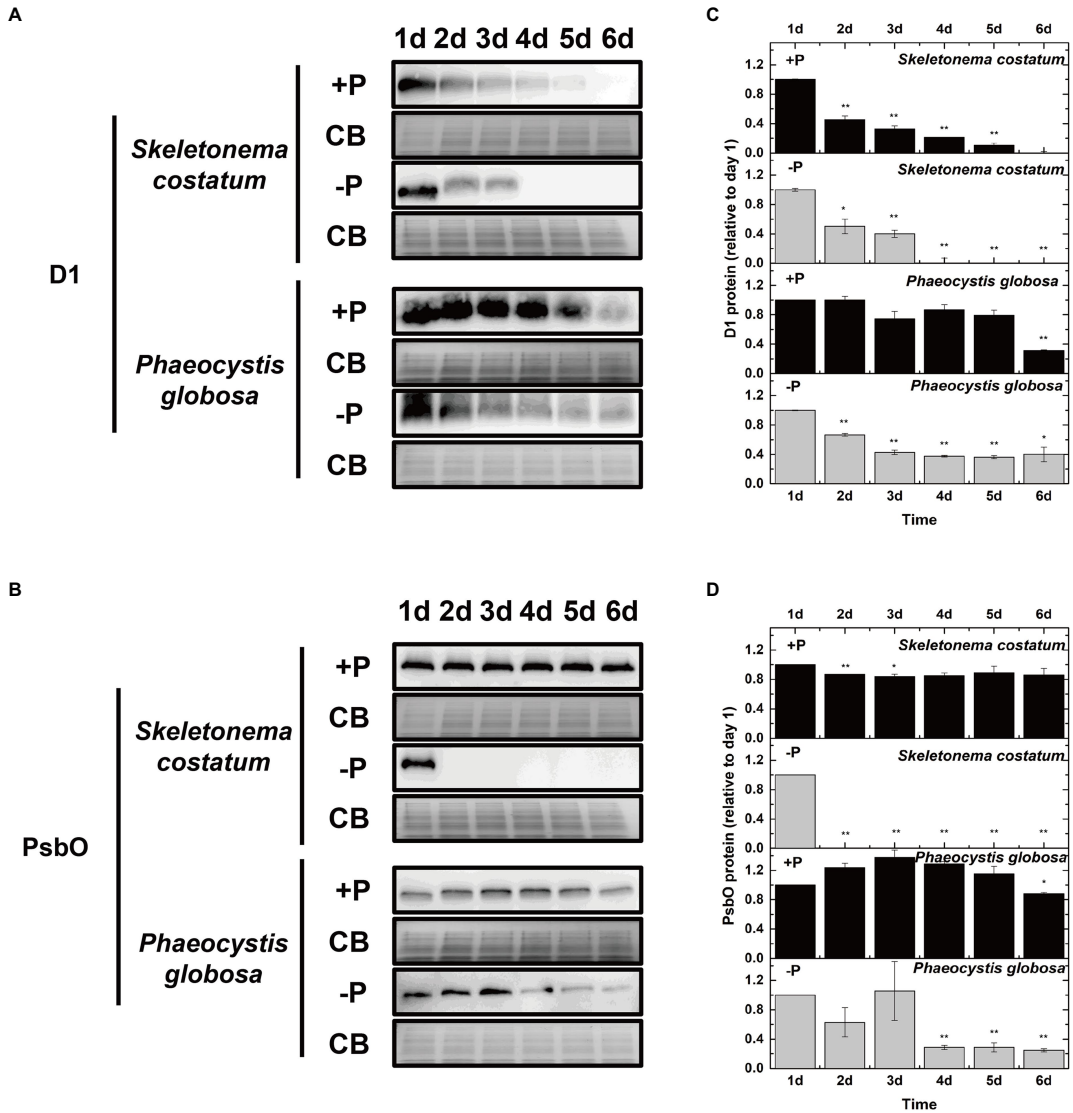
Immunoblot analysis of PSII proteins and stained with Coomassie blue (CB). **(A)** Immunoblot analysis was performed using antibodies specific to D1. **(B)** Immunoblot analysis was performed using an antibody specific to PsbO. **(C)** Ratios of the D1 protein level of P-limited cells were semi-quantitated by the immunoblot analysis relative to that of cells on day 1, which was set to 100% for easy comparison. **(D)** Ratios of the PsbO protein level of P-limited cells were semi-quantitated by the immunoblot analysis relative to that of cells on day 1, which was set to 100% for easy comparison. Error bars show mean ± standard error (SE) of triplicate treatments. Superscripts indicate significant differences between day 1 and other days according to a two-way ANOVA with a *post-hoc* test (^**^p < 0.01, ^*^p < 0.05).

To investigate if the decreased PSII activity under P limitation was associated with the decrease in the PSII protein level, the effects of P limitation on PSII subunits D1 and PsbO were further examined. The decrease in D1 proteins in *S*. *costatum* and *P*. *globosa* in the +P group (Figures 8A,C) could be explained by the gradual consumption of nutrient elements in batch culture experiments (Keren et al., 1997). P limitation results in 100% loss of the D1 protein in *S*. *costatum* in 4 days (Figures 8A,C, *p* < 0.01, ANOVA). However, a 60% decrease was found in the D1 level of *P*. *globosa* under P limitation for 6 days (Figures 8A,C, *p* < 0.05, ANOVA). The PsbO level was stable at 25% of the original value in the later stage of growth under P limitation in *P*. *globosa*. However, a 100% decrease in PsbO protein level was observed in *S*. *costatum* after 2 days of P limitation (Figures 8B,D, *p* < 0.01, ANOVA). Therefore, P limitation resulted in a larger and faster decrease in D1 and PsbO in *S*. *costatum* than in *P*. *globosa*.

### 3.4. P limitation on their photosystem II repair in *Skeletonema costatum* and *Phaeocystis globosa* under P limitation

**Figure 9:**
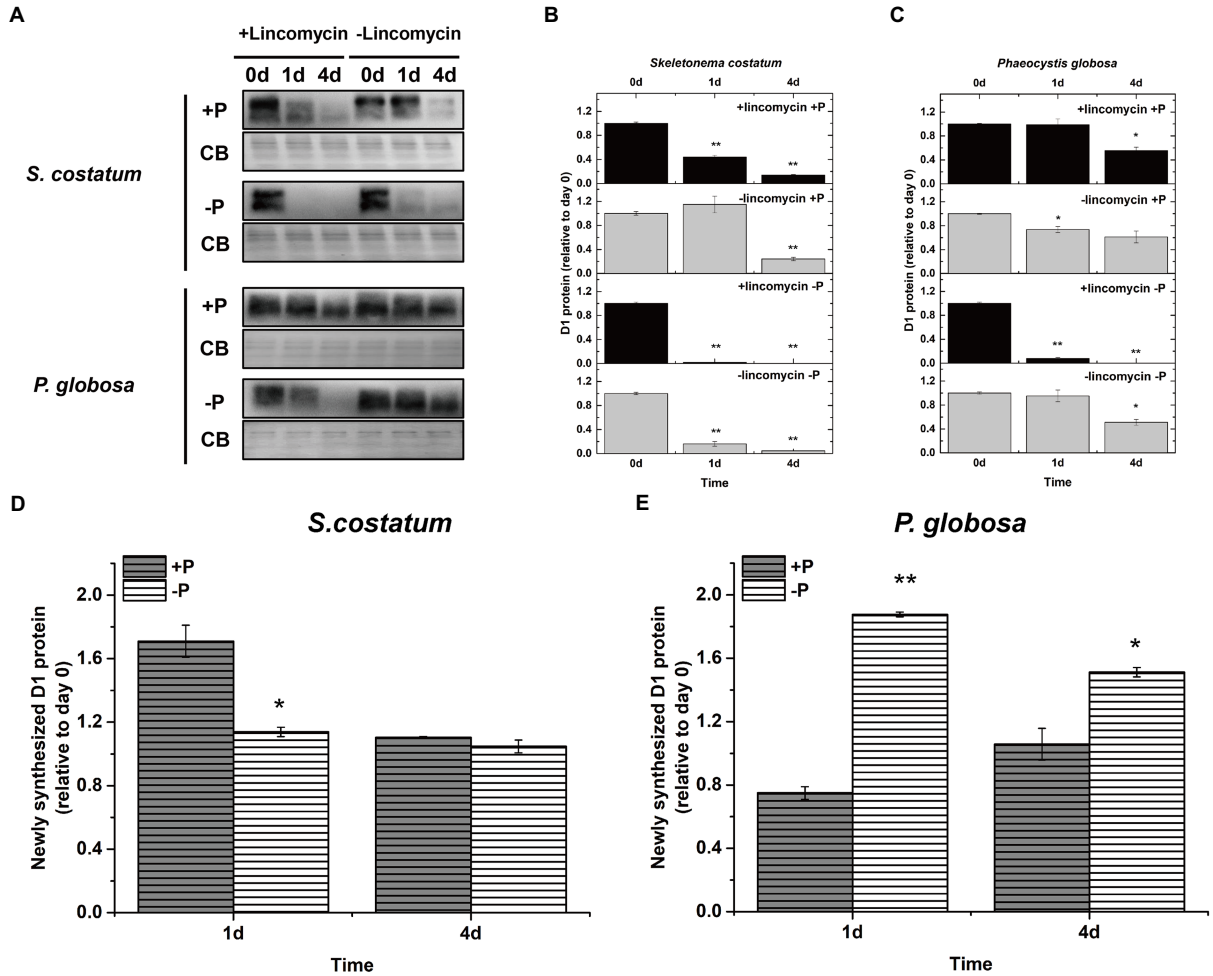
**(A)** Immunoblot analysis of D1 protein levels in *S*. *costatum* and *P*. *globosa* cultures treated with or without lincomycin in +P and −P groups. The blot was stained with Coomassie blue (CB). **(B)** Ratios of the D1 protein level of *S*. *costatum* cells semi-quantitated by the immunoblot analysis relative to that of cells on day 0, which was set to 100% for easy comparison. **(C)** Ratios of the D1 protein level of *P*. *globosa* cells semi-quantitated by the immunoblot analysis relative to that of cells on day 0. **(D)** Newly synthesized D1 protein (relative to day 0) in *S*. *costatum* in +P and −P groups. Newly synthesized D1 in PSII repair was measured as the change in the active D1 content in the absence of lincomycin (PSII repair active) minus the change in the active D1 content in the presence of lincomycin (PSII repair blocked). **(E)** Newly synthesized D1 protein (relative to day 0) in *P*. *globosa* in +P and −P groups. Newly synthesized D1 in PSII repair was measured as the change in the active D1 content in the absence of lincomycin (PSII repair active) minus the change in the active D1 content in the presence of lincomycin (PSII repair blocked). Error bars are mean ± SE of triplicate treatments. Superscripts indicate significant differences according to a two-way ANOVA with a *post-hoc* test (**B,C**: day 0 vs. other days; **D,E**: +P group vs.−P group. ^**^p < 0.01, ^*^p < 0.05).

Cells can repair damaged PSII by replacing photo-damaged proteins in PSII with newly synthesized subunits (Nixon et al., 2010; Komenda et al., 2012). Wu et al. (2012) blocked the counteracting repair processes by adding lincomycin to estimate the newly synthesized D1 protein in PSII repair. To further investigate if stable PSII activity under P limitation in *P*. *globosa* was associated with PSII repair, the newly synthesized D1 was plotted and compared. In both *S*. *costatum* and *P*. *globosa*, treatment with lincomycin elicited a greater drop in the D1 protein level in the absence of P than in the presence of P (Figures 9A–C). The presence of lincomycin resulted in a greater loss of the D1 protein in *S*. *costatum* than in *P*. *globosa* under both P-repleted and P-limited conditions (Figures 9A–C). Without lincomycin, the loss of D1 protein content is still higher in *S*. *costatum* than in *P*. *globosa* under both two conditions (Figures 9A–C). These results indicated that the PSII was impaired more seriously in *S*. *costatum* than in *P*. *globosa* under both conditions. When comparing the newly synthesized D1 protein between P-limited and P-repleted conditions, we found that the newly synthesized D1 protein decreased in *S*. *costatum* (Figure 9D, *p* < 0.05, ANOVA) while increased in *P*. *globosa* after P limitation (Figure 9E, *p* < 0.01, ANOVA), These results suggested that P limitation inhibited the PSII repair in *S*. *costatum* but accelerated it in *P*. *globosa*.

### 3.5. Non-photochemical quenching induction in *Skeletonema costatum* and *Phaeocystis globosa* under P limitation

**Figure 10:**
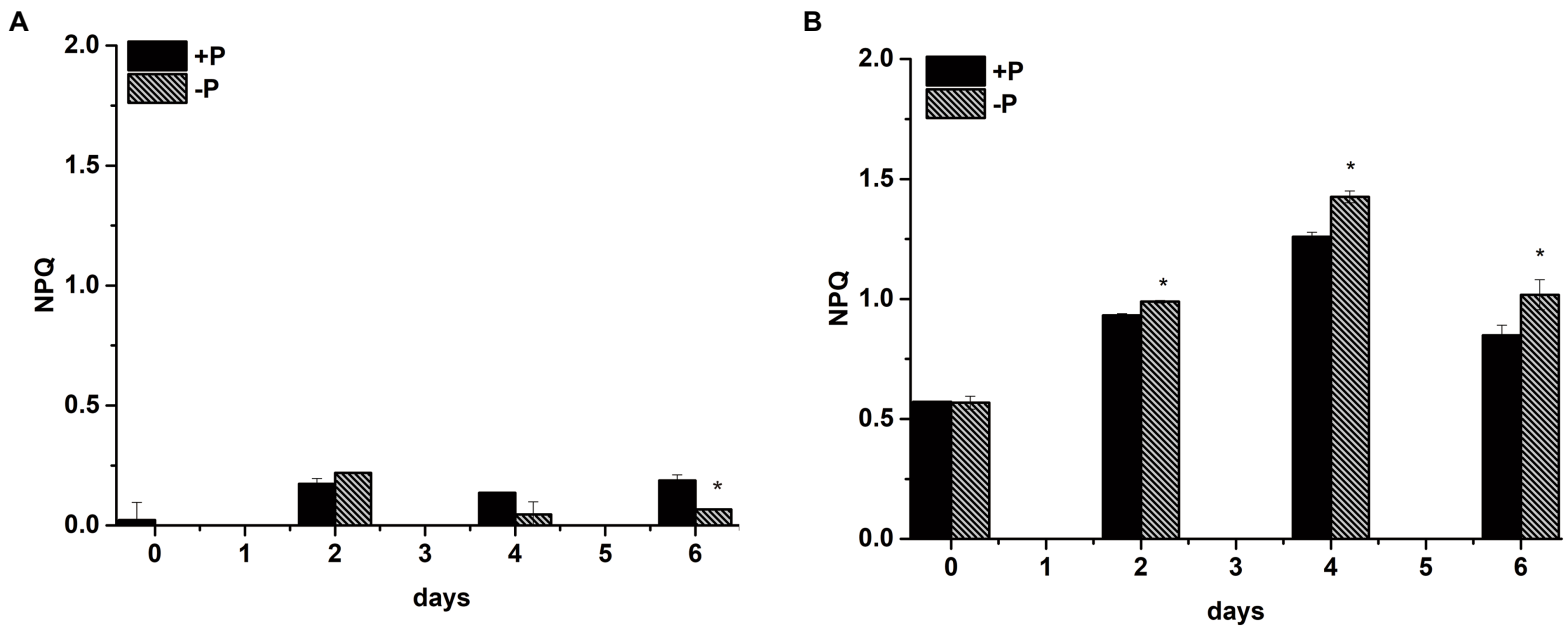
Non-photochemical quenching (NPQ) capacity of *S*. *costatum* **(A)** and *P*. *globosa* **(B)** in +P and −P groups. Error bars show the mean ± standard error (SE) of triplicate treatments. Superscripts indicate significant differences between +P group and −P group according to a two-way ANOVA with a *post-hoc* test (^*^p < 0.05).

To explore whether *S*. *costatum* and *P*. *globosa* enhance their NPQ activity under P limitation to dissipate excess light as heat for protection from potential photo-oxidative damage, the NPQ values were obtained from the slow induction kinetic curves of *S*. *costatum* and *P*. *globosa* in the +P and − P groups for various periods of time (Supplementary Figure S4). The NPQ value of the −P group was significantly higher than that of the +P group, which was expressed in *P*. *globosa* (*p* < 0.05, ANOVA, Figure 10B), indicating that P limitation induced higher NPQ. The NPQ values of *S*. *costatum* were maintained at a low level for various periods, and the increase was not significant in the −P group compared with the +P group (*p* > 0.05, ANOVA, Figure 10A). Notably, *P*. *globosa* had >5-fold higher NPQ relative to the *S*. *costatum* at the same actinic light of 321 μmol photons m^−2^ s^−1^ red light (Figure 10), suggesting that the NPQ capacity of *P*. *globosa* cell was higher than that of *S*. *costatum*. Considering that the NPQ prevents photodamage of the D1 protein in PSII (Lepetit et al., 2012), we suggested that the above more active D1 turnover (Figure 9E) and stable PSII activity (Figure 3B) were due to the higher NPQ capacity *P*. *globosa* under P limitation.

## 4. Discussion

In this study, photosynthetic responses of the diatom *S*. *costatum* and the haptophyte *P*. *globosa* under P limitation were compared. *S*. *costatum* cells could not proliferate after P limitation with the impairment of OEC on the donor side, the inhibition of electron transport from Q_A_^−^ to Q_B_, and the inability to provide electrons downstream of PSII. The fast and complete decrease in D1 and PsbO in *S*. *costatum* were associated with its enhanced photoinhibition. However, when exposed to P limitation, *P*. *globosa* could grow and retain its photosynthetic activity for at least 1 week with minor impairment of PSII function compared with *S*. *costatum*. In particular, the photosynthetic acclimation mechanisms adopted by *P*. *globosa* under P limitation to avoid greater photoinhibition of PSII were highlighted.

### 4.1. Effects of P limitation on growth and their photosystem II activity in *Skeletonema costatum* and *Phaeocystis globosa*

*Phaeocystis globosa* blooms usually occur after diatom blooms in winter to early spring in the SCS and similar trends were also observed in the coastal waters of high latitude North Sea (Lancelot et al., 2005). Karasiewicz et al. (2018) suggested both abiotic and biotic interactions favored *Phaeocystis* blooms with great contribution from the preceding diatoms. The silicate exhaustion has historically been thought to be a major explanation for the appearance of *P*. *globosa* (Lancelot et al., 1987; Reid et al., 1990). The role of P in the transition of phytoplankton communities was reviewed by Lin et al. (2016).

P is an essential nutrient for the growth and proliferation of phytoplankton. P limitation inhibited the growth of *S*. *costatum* remarkably but had no obvious effect on the growth of *P*. *globosa* over a period of 7 days (Figure 1), which strongly suggested that *P*. *globosa* has more tolerance to P limitation compared with *S*. *costatum*. Moreover, the PSII activity of *S*. *costatum* was inhibited by P limitation, whereas *P*. *globosa* maintained photosynthetic activity after P limitation based on the estimation of F_v_/F_m_ and other chlorophyll fluorescence parameters (Figure 2). The obvious growth and photosynthetic activity inhibition of P limitation in *S*. *costatum* are in accordance with those reported previously (Zhang et al., 2016).

In this study, the pre-incubation in P-free medium before this experiment was not done since *S*. *costatum* cannot keep photosynthetic activity after the pre-incubation. P pools associated with cells were identified previously as intracellular P pool and cell surface-adsorbed P pool (Jin et al., 2021). Both *S*. *costatum* and *P*. *globosa* can take up P in excess of its immediate cellular demand under P-sufficient conditions and store it in the form of PolyP, so-called luxury uptake, and break down this PolyP store upon P stress (Schoemann et al., 2001; Solovchenko et al., 2019). As shown in Supplementary Figure S1, both of them have the similar ability to store PolyP. However, the maintenance of growth and photosynthetic activity in *P*. *globosa* under P limitation might be caused by its higher tolerance to the P limitation as it still kept a stable photosynthetic activity after 25 days of P limitation (Supplementary Figure S2).

Photosynthesis can be divided into the light reactions, in which light energy is stored as ATP and NADPH, and the dark reactions, in which the products of the light reactions are used to reduce inorganic C (Green and Durnford, 1996). Phosphorus is an essential element in compounds such as ATP, NADPH, nucleic acids, phospholipids and sugar phosphates, all of which play important roles in photosynthesis. Numerous groups have demonstrated that P limitation causes a decline in photosynthesis in a number of different ways (Heraud et al., 2005; Shelly et al., 2005). It was proposed that a decreased supply of Pi reduced the ATP synthesis/levels, thereby, reducing both phosphorylation and CO_2_ assimilation (Lewis et al., 1994; Campbell and Sage, 2006). Other studies also reported that the photosynthetic machinery composition or electron transport activity was also affected under P-limited condition (Van Rensen and Vredenberg, 2009; Carstensen et al., 2018). Wykoff et al. (1998) reported that P deprivation seriously inhibited the PSII activity but not that of PSI in C. *reinhardtii*. In this study, we mainly focused on the performance of PSII.

### 4.2. Different performance of P limitation on their photosystem II in *Skeletonema costatum* and *Phaeocystis globosa* under P limitation

P limitation on their photosystem II, a multiprotein complex present in the thylakoid membranes of oxygenic photosynthetic organisms, is the center of photosynthesis that uses light to drive water oxidation and PQ reduction (Iwata and Barber, 2004). PSII is artificially divided into the donor sides (OEC), reaction centers, and acceptor sides. The donor sides split water and evolve oxygen (Wilson and Jain, 2018). The reaction center of PSII carries out photochemical reactions, including the primary charge separation and subsequent electron transfer from water to PQ (Semenov et al., 2011). On the electron acceptor side of PSII, Q_A_ (a one-electron acceptor PQ) takes up one electron from Phe_D1_^−^ (a pheophytin molecule) and transfers it to Q_B_ (a two-electron acceptor PQ; Zhang et al., 2017). In this study, various chlorophyll fluorescence tools were used to examine the physiological response to P limitation in and around PSII.

P limitation inhibited electron transfer from Q_A_^−^ to Q_B_ in *S*. *costatum* and *P*. *globosa*, which was suggested by the increase in the fluorescence intensity of phase J (Figures 4A,B), the increase in the decay half-life of the fast phase obtained from the Q_A_^−^ reoxidation kinetics test (Table 1) and the increase in the proportion of PSII_X_ centers (Figures 7A,B). The increase in PSII_X_ centers, also called Q_B_-nonreducing PSII centers, under P limitation was also observed in *Chlamydomonas reinhardtii*, *Chlorella*, and *Scenedesmus* (Wykoff et al., 1998; Dao and Beardall, 2016; Markou et al., 2017). These results provide circumstantial evidence that both *S*. *costatum* and *P*. *globosa* accumulate inactive PSII unable to reduce PQ under P limitation, which might be a way to acclimate to abiotic stress including P starvation. Previous studies have found that these centers are resistant to photoinhibition and capable of facilitating excess excitation energy dissipation for photosynthetic organisms under abiotic stress conditions (Neale and Melis, 1990; Hill and Ralph, 2006). A higher concentration of Q_B_-nonreducing PSII centers was also shown in the triazine-resistant plant *Chenopodium album* (Van Rensen and Vredenberg, 2009).

The impairment of the donor side in PSII by P limitation has been reported in wheat, rice, citrus, and other higher plants (Lin et al., 2009; Veronica et al., 2017; Meng et al., 2021). From the Q_A_^−^ reoxidation kinetics test in the presence of DCMU (Table 1), the donor side of PSII in *S*. *costatum* was damaged after exposure to P limitation for 2 days. These results are in accordance with the observed positive K-step in the OJIP transients (Figure 4C) and a sharp decrease in RC/CS_0_ (Figure 5A) of *S*. *costatum* under P limitation. However, because the changes in these parameters resulting from P limitation were small or even negligible in *P*. *globosa*, the donor side of PSII in *P*. *globosa* might be damaged by P limitation but not seriously. The fact that P limitation caused more serious damage to OEC in *S*. *costatum* than in *P*. *globosa* was further confirmed by the different PsbO expression patterns in the two species under P limitation. PsbO is an extrinsic protein that plays a crucial role in the structure and function of the OEC of PSII (Popelkova and Yocum, 2011). Jain et al. (2005) reported that P limitation suppresses the expression of *psbO* (PsbO protein encoding gene) in *Arabidopsis thaliana*, thus impairing the structural integrity of OEC and consequently its function. In this study, a 100% decrease of PsbO in *S*. *costatum* was observed after exposure to P limitation for 2 days, which was in accordance with the physiological result that P limitation resulted in a serious impairment of the donor side in PSII in *S*. *costatum*. However, PsbO in *P*. *globosa* showed a slow decrease and remained at a certain level in the later stage of culture under P limitation (Figures 8B,D), reflecting smaller and slighter damage of OEC caused by P limitation in *P*. *globosa*.

Impairment of PSII finally led to the inhibition of electron transport to PSI. P limitation inhibiting electron transport to PSI has been demonstrated in barley (Carstensen et al., 2018) and dinoflagellate *Peridinium bipes* revealed by the decrease in φE_0_ and RE_0_/RC (Yang et al., 2020). P limitation resulted in the gradual and significant decline in φE_0_ and RE_0_/RC values in *S*. *costatum* (Figure 5A) rather than in *P*. *globosa*. These results confirmed that the P limitation only leads to the inhibition of electron transport after Q_A_^−^ to the PSI electron acceptor side of *S*. *costatum*. However, the electron transport to PSI in *P*. *globosa* was not affected by P limitation (Figure 5B).

Thus, P limitation caused serious PSII damage with obvious impairment of both donor and acceptor sides of PSII in *S*. *costatum*; *P*. *globosa* under P limitation showed minor impairment of OEC of PSII compared with *S*. *costatum* and no obvious inhibition of electron transport to PSI. D1 constitutes the core of the PSII reaction center. PSII inactivation in *C*. *reinhardtii* subjected to P deprivation is the result of enhanced photoinhibition shown by enhanced D1 degradation (Wykoff et al., 1998; Malnoe et al., 2014). In this study, P limitation resulted in a much faster and larger decrease in D1 in *S*. *costatum* than in *P*. *globosa*, which is associated with the worse performance of PSII in the former than in the latter.

### 4.3. Regulation of D1 turnover and non-photochemical quenching in *Skeletonema costatum* and *Phaeocystis globosa* under P limitation

PSII repair involves partial disassembly of the damaged complex, selective proteolytic degradation, and replacement of the damaged subunit (predominantly the D1 reaction center subunit) by *de novo* synthesized copy and reassembly. Turnover of the D1 protein is required for PSII repair and restoration of PSII photochemical activity after photoinhibition (Nixon et al., 2010). P limitation causes photodamage of PSII in *S*. *costatum* by not only directly accelerating photoinactivation but also inhibiting D1 restoration. Inhibited D1 restoration under P starvation has also been reported in *D*. *tertiolecta* (Heraud et al., 2005). This may be because protein synthesis (protein turnover) is energetically costly, requiring 10.8 ATP per peptide bond, and P limitation often causes a decline in nucleotides such as ATP and GTP (Murata and Nishiyama, 2018). In contrast, *P*. *globosa* under P limitation showed increased D1 restoration obtaining a smaller decrease in the D1 level compared with *S*. *costatum*. It remains to be explained how *P*. *globosa* synthesizes a high amount of D1 under P limitation. *P*. *globosa* still maintains active D1 synthesis while ceasing synthesis of most other proteins, including the photosynthetic apparatus subunits in P limitation (Feng et al., 2015). Under unfavorable P-limited conditions, *P*. *globosa* invests much more energy in optimization and maintaining of light harvesting (such as *de novo* D1 synthesis), but not under P-replete conditions. This flexibility of *P*. *globosa* provides an advantage in the fluctuating environment. Similarly, the acceleration of D1 synthesis has been reported in *Emiliania huxleyi* under short-term P depletion (Loebl et al., 2010) and in the cyanobacterium *Synechocystis* sp. PCC 6803 under oxidative stress (Yu et al., 2014). Loebl et al. (2010) also found that the superior ability of PSII repair in *E*. *huxleyi* helps maintain significant PSII function over at least 38 days under nitrogen depletion. Undoubtedly, *P*. *globosa*, but not *S*. *costatum*, could counteract photoinactivation by the acceleration of PSII repair, thus maintaining stable photosynthetic activity under P limitation.

The photosynthetic apparatus showed higher resistance in *P*. *globosa*, which might also be caused by more effective energy dissipative mechanisms. NPQ allows for the safe dissipation of excess excitation energy as heat in light-harvesting antenna complexes (LHCs) of PSII and prevents over-reduction in the electron transport chain that may lead to reactive oxygen species (ROS) production (Muller et al., 2001). It thereby prevents photodamage of the D1 protein in PSII, which is the primary target of ROS-generated oxidative stress (Wu et al., 2011; Lepetit et al., 2012). The main part of NPQ in microalgae is called qE, which relies on the interconversion of specific pigments by the xanthophyll cycle. The xanthophyll cycle in green and brown algae is composed of violaxanthin, antheraxanthin, and zeaxanthin (VAZ cycle), while in dinoflagellates, diatoms, and haptophytes, it consists of diadinoxanthin and diatoxanthin (Dd-Dt cycle; Eisenstadt et al., 2008; Goss and Jakob, 2010).

Both *P*. *globosa* and *S*. *costatum* possess the Dd-Dt cycle and can promote NPQ (Lavaud et al., 2002; Harris et al., 2005). From the induction kinetics of NPQ (Figure 10B), *P*. *globosa* cells could enhance their NPQ activity as an adaptive strategy under P limitation. This can be explained as follows: P limitation reduces the orthophosphate concentration in the chloroplast stroma and causes the inhibition of ATP synthase activity. Consequently, protons accumulate in the thylakoids and cause a high trans-thylakoidal proton gradient activating the NPQ mechanism (Lavaud et al., 2004; Lavaud and Lepetit, 2013). Enhancement of NPQ is also an adaptive strategy under P limitation in the dinoflagellate *Karlodinium veneficum* (Cui et al., 2017), diatom *Thalassiosira pseudonana* (Li et al., 2021), and chlorophytes *D*. *tertiolecta* (Petrou et al., 2008) and *C*. *reinhardtii* (Wykoff et al., 1998). Indeed, the induction of LHCSR (Light Harvesting Complex Stress Related), which controls the recruitment of NPQ in *C*. *reinhardtii*, has also been reported under P deprivation (Moseley et al., 2006). Thus, NPQ, as a photosynthetic mechanism to cope with P limitation, plays an important role in maintaining the balance of the energy budget in P-limited *P*. *globosa* cells.

Su et al. (2012) and Lavaud et al. (2016) pointed out that *S*. *costatum* had limited capacity for induction of NPQ. Previous studies suggested two molecular explanations for the low NPQ in *S*. *costatum* (Lavaud and Lepetit, 2013): (1) a lower amount of Dt molecules involved in NPQ, and (2) lower capacity to form large functionally disconnected oligomeric fucoxanthin–chlorophyll protein complexes caused by the different organization and composition of LHC of PSII. Ultimately, the inability of *S*. *costatum* to promote a similarly strong NPQ as in *P*. *globosa* leads to a higher susceptibility of *S*. *costatum* to photoinhibition during exposure to P limitation.

### 4.4. Photosynthetic responses to P limitation are associated with the survival, dominance, and succession of marine microalgae under P limitation

P limitation was considered one of the factors favoring the population shift from diatoms or other early succession species to late succession species such as dinoflagellates, haptophytes, and pelagophytes, which are better adapted to this new set of environmental conditions (Lin et al., 2016). Considering that photosynthesis is the most important driver of cell growth and other physiological processes in phytoplankton, these different photosynthetic responses in algal bloom species may also contribute to their collapse or well-being under P limitation, thus affecting the transition of phytoplankton communities from early succession species to late succession species.

**Figure 11:**
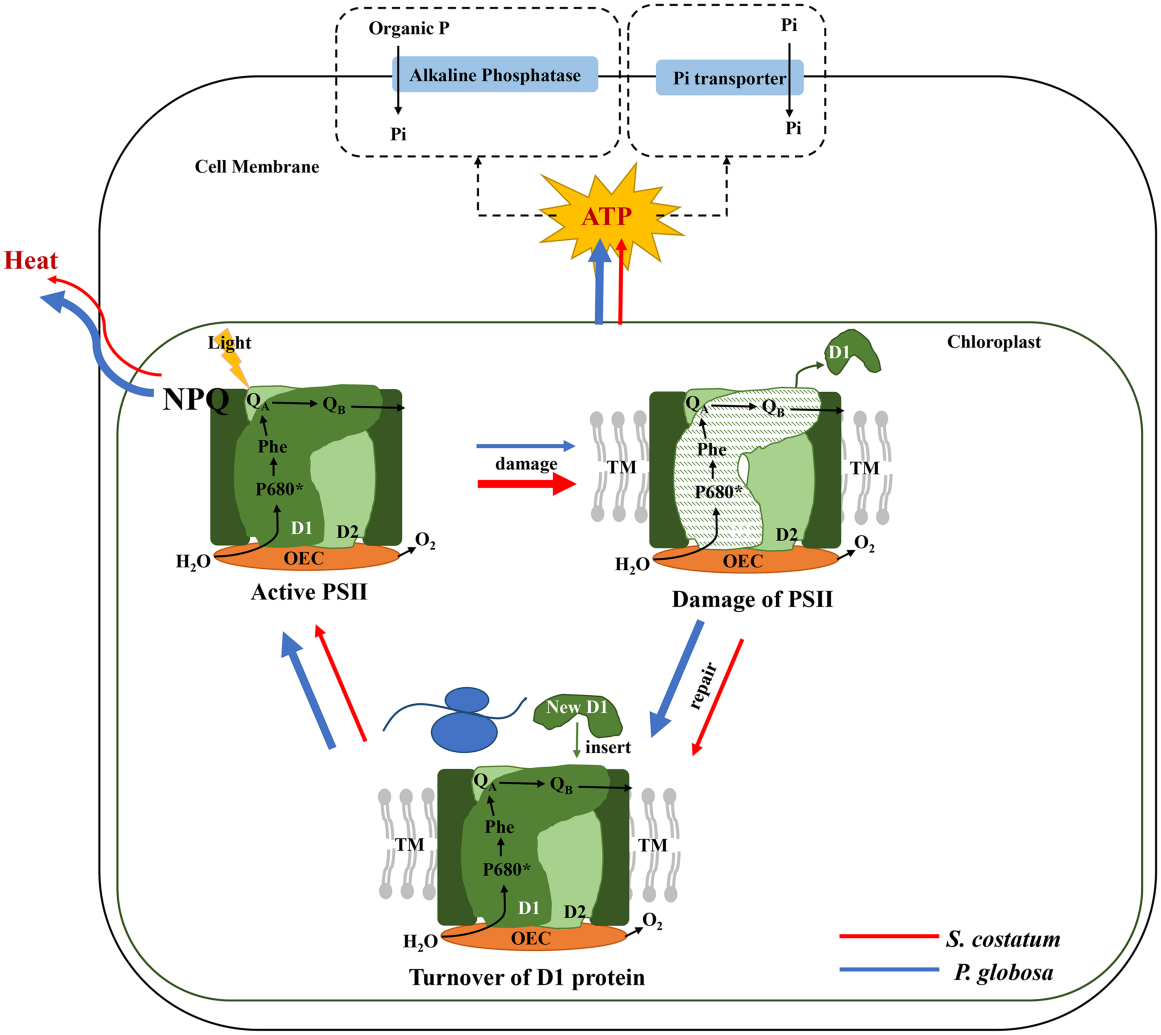
The photosynthetic responses of *S*. *costatum* (red line) and *P*. *globosa* (blue line) under P limitation (the thickness of the line indicates the strength of the effect).

Liu et al. (2013) suggested that the photosynthetic capacity of *S*. *costatum* was limited by P limitation, and F_v_/F_m_ decreased in *S*. *costatum* under P limitation (Li and Sun, 2016). This study demonstrated that P limitation seriously impaired PSII in *S*. *costatum*, and this species did not have a strong ability to induce high NPQ and accelerate the PSII repair cycle to decrease photosynthetic impairment resulting from P limitation. From the results of the present study, *P*. *globosa* could maintain relatively stable photosynthetic activity under P limitation by inducing high NPQ and accelerating D1 restoration (Figure 11). The photosynthesis activity might affect the P limitation tolerance because the energy sources for Pi uptake and the synthesis of APase in microalgae are mostly derived from photosynthesis (Pandey, 2006; Gorbunov and Falkowski, 2022). The superior photosynthetic performance of *P*. *globosa* under P limitation was undoubtedly conducive to its well-being, which is consistent with previous ecological investigations (Wang K. et al., 2021; Wang X. D. et al., 2021).

The succession of other species under P limitation has also been reported to be closely correlated with photosynthetic responses. An apparent bloom-forming species succession with the shifting from diatom blooms (mainly *S*. *costatum*) in the early spring to long-lasting and large-scale dinoflagellate blooms dominated by *Prorocentrum donghaiense* was observed in the coastal waters of the East China Sea (Lu et al., 2022), which is also reported to be in accordance with the different response of *S*. *costatum* and *P*. *donghaiense* to the phosphate exhaustion (Li et al., 2011; Ou et al., 2020). Although the study about the comparison of photosynthesis response to P limitation of these two strains is still limited, it is clear that *P*. *donghaiense* had higher photosynthetic activity and potential than *S*. *costatum* under eutrophic but relatively P-limited conditions according to Li and Sun (2016). P limitation is also one of the critical factors allowing the coccolithophorid *E*. *huxleyi* to bloom after diatom blooms in temperate coastal and oceanic areas (Tyrrell and Merico, 2004; Lessard et al., 2005; Oguz and Merico, 2006), although these conditions are not mandatory for *E*. *huxleyi* blooms (Lessard et al., 2005). Zhao et al. (2015) reported that P limitation resulted in greater damage to the photosynthetic apparatus in *Phaeodactylum tricornutum*, while *E*. *huxleyi* under P limitation showed increased xanthophyll cycle pigment accumulation and more active transformation from diadinoxanthin to diatoxanthin than *P*. *tricornutum*. Hence, the different photosynthetic responses to P limitation in some algal bloom species might be associated with their survival, dominance, and succession under P limitation.

## 5. Conclusion

The photosynthetic responses for the different tolerance to P limitation in the diatom *S*. *costatum* and the haptophyte *P*. *globosa* were investigated and compared. When exposed to P limitation, *S*. *costatum* could not proliferate with obvious impairment of both donor and acceptor sides of PSII, and the inhibition of electron transport to PSI. The fast and complete decrease in the D1 and PsbO levels of *S*. *costatum* were associated with its enhanced photoinhibition. On the contrary, *P*. *globosa* could decrease susceptibility to photoinactivation of PSII with minor impairment of the donor side and thus the reaction center of PSII compared with *S*. *costatum*. By the induction of NPQ and a faster D1 turnover in PSII repair, *P*. *globosa* avoided further photoinhibition and maintained its stable photosynthetic capacity under P limitation. In addition, although not specifically investigated in this study, the different photosynthetic responses to P limitation in some algal bloom species might be crucial to explain their survival, dominance, and succession under P limitation. Overall, these findings can enhance understanding of the effect on the photosynthetic apparatus of PSII by P limitation in marine algae, elucidate the photosynthetic responses for superior tolerance to P limitation in *P*. *globosa* than in *S*. *costatum* when cells undergo P limitation, and provide additional evidence explaining the transition of phytoplankton communities from diatom to late succession haptophytes.

## Data availability statement

The original contributions presented in the study are included in the article/Supplementary material, further inquiries can be directed to the corresponding author.

## Author contributions

LS conceived and initiated the project. XC performed all the experiments and analyzed the data. JZ and LS designed and supervised all the experiments. LZ helped to isolate the strains used in the manuscript. JL provided resources and methodology. JZ and XC wrote the article. All authors provided comments and approved the final version of the article.

## Funding

This work was supported by the Funds for International Cooperation and Exchange of the National Natural Science Foundation of China (Grant No. 3201101157), National Key Research and Development Program of China (Grant No. 2016YFE0202100), and National Natural Science Foundation of China (Grant No. 32270136).

## Conflict of interest

The authors declare that they have no known competing financial interests or personal relationships that could have appeared to influence the work reported in this paper.

## Publisher’s note

All claims expressed in this article are solely those of the authors and do not necessarily represent those of their affiliated organizations, or those of the publisher, the editors and the reviewers. Any product that may be evaluated in this article, or claim that may be made by its manufacturer, is not guaranteed or endorsed by the publisher.

## References

[ref1] Allen M. D. Kropat J. Tottey S. Del Campo J. A. Merchant S. S. (2007). Manganese deficiency in *Chlamydomonas* results in loss of photosystem II and MnSOD function, sensitivity to peroxides, and secondary phosphorus and iron deficiency. Plant Physiol. 143, 263–277. doi: 10.1104/pp.106.088609, PMID: 17085511 PMC1761973

[ref2] Armbrust E. V. (2009). The life of diatoms in the world's oceans. Nature 459, 185–192. doi: 10.1038/nature08057, PMID: 19444204

[ref3] Beauchemkin R. Gauthier A. Harnois J. Boisvert S. Govindachary S. Carpentier R. (2007). Spen-nine and spermidine inhibition of photosystem II: disassembly of the oxygen evolving complex and consequent perturbation in electron donation from Tyrz to P680(+) and the quinone acceptors Q(a)(−) to Q(B). BBA-Bioenergetics 1767, 905–912. doi: 10.1016/j.bbabio.2007.04.001, PMID: 17511958

[ref4] Bilger W. Bjorkman O. (1990). Role of the xanthophyll cycle in photoprotection elucidated by measurements of light-induced absorbency changes, fluorescence and photosynthesis in leaves of *Hedera canariensis*. Photosynth. Res. 25, 173–185. doi: 10.1007/Bf00033159, PMID: 24420348

[ref5] Burson A. Stomp M. Akil L. Brussaard C. P. D. Huisman J. (2016). Unbalanced reduction of nutrient loads has created an offshore gradient from phosphorus to nitrogen limitation in the North Sea. Limnol. Oceanogr. 61, 869–888. doi: 10.1002/lno.10257

[ref7] Cadee G. C. Hegeman J. (2002). Phytoplankton in the Marsdiep at the end of the 20th century; 30 years monitoring biomass, primary production, and *Phaeocystis* blooms. J. Sea Res. 48, 97–110. doi: 10.1016/S1385-1101(02)00161-2

[ref8] Campbell C. D. Sage R. F. (2006). Interactions between the effects of atmospheric CO_2_ content and P nutrition on photosynthesis in white lupin (*Lupinus albus* L.). Plant Cell Environ. 29, 844–853. doi: 10.1111/j.1365-3040.2005.01464.x 17087468

[ref9] Cao J. Govindjee M. (1990). Chlorophyll-a fluorescence transient as an indicator of active and inactive photosystem II in thylakoid membranes. Biochim. Biophys. Acta 1015, 180–188. doi: 10.1016/0005-2728(90)90018-Y, PMID: 2404518

[ref10] Carstensen A. Herdean A. Schmidt S. B. Sharma A. Spetea C. Pribil M.. (2018). The impacts of phosphorus deficiency on the photosynthetic electron transport chain. Plant Physiol. 177, 271–284. doi: 10.1104/pp.17.01624, PMID: 29540590 PMC5933119

[ref11] Chan A. T. (1980). Comparative physiological study of marine diatoms and dinoflagellates in relation to irradiance and cell-size 2: Relationship between photosynthesis, growth, and carbon/chlorophyll *a* ratio. J. Phycol. 16, 428–432. doi: 10.1111/j.1529-8817.1980.tb03056.x

[ref12] Chen T. G. Chen Z. R. Tang C. L. Song D. (2009). Trend of mean winter sea surface temperature (SST) along eastern Guangdong coastal waters during 1898-2009. Guangdong Meteorol. 4, 1–3. doi: 10.3969/j.issn.1007-6190.2009.04.001

[ref13] Chen B. H. Kang W. Xu D. Hui L. (2021). Long-term changes in red tide outbreaks in Xiamen Bay in China from 1986 to 2017. Estuar. Coast. Shelf 249:107095. doi: 10.1016/j.ecss.2020.107095

[ref14] Chen F. J. Xu N. Jiang T. J. Wang Y. Wang Z. H. Qi Y. Z. (1999). A report of *Phaeocystis globosa* bloom in coastal water of Southeast China. J. Jinan Univ. 20, 124–129.

[ref15] Chylla R. A. Whitmarsh J. (1989). Inactive photosystem II complexes in leaves–turnover rate and quantitation. Plant Physiol. 90, 765–772. doi: 10.1104/pp.90.2.765, PMID: 16666841 PMC1061794

[ref16] Cui Y. D. Zhang H. Lin S. J. (2017). Enhancement of non-photochemical quenching as an adaptive strategy under phosphorus deprivation in the dinoflagellate *Karlodinium veneficum*. Front. Microbiol. 8:43. doi: 10.1016/j.ecoenv.2018.04.043, PMID: 28360892 PMC5350143

[ref17] Dao L. H. T. Beardall J. (2016). Effects of lead on two green microalgae *chlorella* and *Scenedesmus*: photosystem II activity and heterogeneity. Algal Res. 16, 150–159. doi: 10.1016/j.algal.2016.03.006

[ref18] Dau H. (1994). Molecular mechanisms and quantitative models of variable photosystem II fluorescence. Photochem. Photobiol. 60, 1–23. doi: 10.1016/j.algal.2016.03.006

[ref19] Deng J. M. Qin B. Q. Paerl H. W. Zhang Y. L. Wu P. Ma J. R.. (2014). Effects of nutrients, temperature and their interactions on spring phytoplankton community succession in Lake Taihu, China. PLoS One 9:e113960. doi: 10.1371/journal.pone.0113960, PMID: 25464517 PMC4252073

[ref21] Egge J. K. (1998). Are diatoms poor competitors at low phosphate concentrations? J. Mar. Syst. 16, 191–198. doi: 10.1016/S0924-7963(97)00113-9

[ref22] Eisenstadt D. Ohad I. Keren N. Kaplan A. (2008). Changes in the photosynthetic reaction center II in the diatom *Phaeodactylum tricornutum* result in non-photochemical fluorescence quenching. Environ. Microbiol. 10, 1997–2007. doi: 10.1111/j.1462-2920.2008.01616.x, PMID: 18397307

[ref23] Escaravage V. Peperzak L. Prins T. C. Peeters J. C. H. Joordens J. C. A. (1995). The development of a *Phaeocystis* bloom in a mesocosm experiment in relation to nutrients, irradiance and coexisting algae. Ophelia 42, 55–74. doi: 10.1080/00785326.1995.10431497

[ref24] Falkowski P. G. Barber R. T. Smetacek V. (1998). Biogeochemical controls and feedbacks on ocean primary production. Science 281, 200–206. doi: 10.1126/science.281.5374.200, PMID: 9660741

[ref25] Feng T. Y. Yang Z. K. Zheng J. W. Xie Y. Li D. W. Murugan S. B.. (2015). Examination of metabolic responses to phosphorus limitation via proteomic analyses in the marine diatom *Phaeodactylum tricornutum*. Sci. Rep. 5:373. doi: 10.1038/srep10373, PMID: 26020491 PMC4446992

[ref26] Ghyoot C. Gypens N. Flynn K. J. Lancelot C. (2015). Modeling alkaline phosphatase activity in microalgae under orthophosphate limitation: the case of *Phaeocystis globosa*. J. Plankton Res. 37, 869–885. doi: 10.1038/srep10373

[ref27] Gorbunov M. Y. Falkowski P. G. (2022). Using chlorophyll fluorescence to determine the fate of photons absorbed by phytoplankton in the World's oceans. Annu. Rev. Mar. Sci. 14, 213–238. doi: 10.1146/annurev-marine-032621-122346, PMID: 34460315

[ref28] Goss R. Jakob T. (2010). Regulation and function of xanthophyll cycle-dependent photoprotection in algae. Photosynth. Res. 106, 103–122. doi: 10.1007/s11120-010-9536-x, PMID: 20224940

[ref29] Green B. R. Durnford D. G. (1996). The chlorophyll-carotenoid proteins of oxygenic photosynthesis. Annu. Rev. Plant Physiol. Plant Mol. Biol. 47, 685–714. doi: 10.1146/annurev.arplant.47.1.685, PMID: 15012305

[ref30] Gypens N. Lacroix G. Lancelot C. (2007). Causes of variability in diatom and *Phaeocystis* blooms in Belgian coastal waters between 1989 and 2003: a model study. J. Sea Res. 57, 19–35. doi: 10.1016/j.seares.2006.07.004

[ref31] Haldimann P. Fracheboud Y. Stamp P. (1995). Carotenoid composition in Zea mays developed at sub-optimal temperature and different light intensities. Physiol. Plantar. 95, 409–414. doi: 10.1111/j.1399-3054.1995.tb00856.x

[ref32] Harris G. N. Scanlan D. J. Geider R. J. (2005). Acclimation of *Emiliania huxleyi* (Prymnesiophyceae) to photon flux density. J. Phycol. 41, 851–862. doi: 10.1111/j.1529-8817.2005.00109.x

[ref34] Heraud P. Roberts S. Shelly K. Beardall J. (2005). Interactions between UV-B exposure and phosphorus nutrition. II. Effects on rates of damage and repair. J. Phycol. 41, 1212–1218. doi: 10.1111/j.1529-8817.2005.00149.x

[ref35] Hill R. Ralph P. J. (2006). Photosystem II heterogeneity of in hospite zooxanthellae in scleractinian corals exposed to bleaching conditions. Photochem. Photobiol. 82, 1577–1585. doi: 10.1562/2006-04-13-Ra-871, PMID: 16961432

[ref36] Huang K. X. Feng Q. L. Zhang Y. Ou L. J. Cen J. Y. Lu S. H.. (2020). Comparative uptake and assimilation of nitrate, ammonium, and urea by dinoflagellate *Karenia mikimotoi* and diatom *Skeletonema costatum* s.l. in the coastal waters of the East China Sea. Mar. Pollut. Bull. 155:111200. doi: 10.1016/j.marpolbul.2020.111200, PMID: 32469790

[ref37] Iwata S. Barber J. (2004). Structure of photosystem II and molecular architecture of the oxygen-evolving center. Curr. Opin. Struct. Biol. 14, 447–453. doi: 10.1016/j.sbi.2004.07.002, PMID: 15313239

[ref38] Jahnke J. (1989). The light and temperature dependence of growth rate and elemental composition of *Phaeocystis globosa* Scherffel and *Phaeocystis pouchetii* (Har) Lagerh in batch cultures. Neth. J. Sea Res. 23, 15–21. doi: 10.1016/0077-7579(89)90038-0

[ref39] Jain A. Cao A. Karthikeyan A. S. Baldwin J. C. Raghothama K. G. (2005). Phosphate deficiency suppresses expression of light-regulated psbO and psbP genes encoding extrinsic proteins of oxygen-evolving complex of PSII. Curr. Sci. 89, 1592–1596.

[ref40] Jin J. Liu S. M. Ren J. L. (2021). Phosphorus utilization by phytoplankton in the Yellow Sea during spring bloom: cell surface adsorption and intracellular accumulation. Mar. Chem. 231:103935. doi: 10.1016/j.marchem.2021.103935

[ref41] Karasiewicz S. Breton E. Lefebvre A. Farinas T. H. Lefebvre S. (2018). Realized niche analysis of phytoplankton communities involving HAB: *Phaeocystis* spp. as a case study. Harmful Algae 72, 1–13. doi: 10.1016/j.hal.2017.12.005, PMID: 29413380

[ref42] Keren N. Berg A. VanKan P. J. M. Levanon H. Ohad I. (1997). Mechanism of photosystem II photoinactivation and D1 protein degradation at low light: the role of back electron flow. P. Natl. Acad. Sci. U. S. A. 94, 1579–1584. doi: 10.1073/pnas.94.4.1579, PMID: 11038602 PMC19834

[ref43] Komenda J. Sobotka R. Nixon P. J. (2012). Assembling and maintaining the photosystem II complex in chloroplasts and cyanobacteria. Curr. Opin. Plant Biol. 15, 245–251. doi: 10.1016/j.pbi.2012.01.017, PMID: 22386092

[ref44] Koppelle S. Lopez-Escardo D. Brussaard C. P. D. Huisman J. Philippart C. J. M. Massana R.. (2022). Mixotrophy in the bloom-forming genus *Phaeocystis* and other haptophytes. Harmful Algae 117:102292. doi: 10.1016/j.hal.2022.102292, PMID: 35944956

[ref46] Lancelot C. Billen G. Sournia A. Weisse T. Colijn F. Veldhuis M. J. W.. (1987). *Phaeocystis* blooms and nutrient enrichment in the continental coastal zones of the North-Sea. Ambio 16, 38–46.

[ref47] Lancelot C. Spitz Y. Gypens N. Ruddick K. Becquevort S. Rousseau V.. (2005). Modeling diatom and *Phaeocystis* blooms and nutrient cycles in the southern bight of the North Sea: the MIRO model. Mar. Ecol. Prog. Ser. 289, 63–78. doi: 10.3354/meps289063

[ref48] Lavaud J. Lepetit B. (2013). An explanation for the inter-species variability of the photoprotective non-photochemical chlorophyll fluorescence quenching in diatoms. BBA-Bioenergetics 1827, 294–302. doi: 10.1016/j.bbabio.2012.11.012, PMID: 23201475

[ref49] Lavaud J. Rousseau B. Etienne A. L. (2004). General features of photoprotection by energy dissipation in planktonic diatoms (Bacillariophyceae). J. Phycol. 40, 130–137. doi: 10.1046/j.1529-8817.2004.03026.x

[ref50] Lavaud J. Rousseau B. van Gorkom H. J. Etienne A. L. (2002). Influence of the diadinoxanthin pool size on photoprotection in the marine planktonic diatom *Phaeodactylum tricornutum*. Plant Physiol. 129, 1398–1406. doi: 10.1104/pp.002014, PMID: 12114593 PMC166533

[ref51] Lavaud J. Six C. Campbell D. A. (2016). Photosystem II repair in marine diatoms with contrasting photophysiologies. Photosynth. Res. 127, 189–199. doi: 10.1007/s11120-015-0172-3, PMID: 26156125

[ref52] Lavergne J. Leci E. (1993). Properties of inactive photosystem II centers. Photosynth. Res. 35, 323–343. doi: 10.1007/Bf00016563, PMID: 24318762

[ref53] Lepetit B. Goss R. Jakob T. Wilhelm C. (2012). Molecular dynamics of the diatom thylakoid membrane under different light conditions. Photosynth. Res. 111, 245–257. doi: 10.1007/s11120-011-9633-5, PMID: 21327535

[ref54] Lessard E. J. Merico A. Tyrrell T. (2005). Nitrate: phosphate ratios and *Emiliania huxleyi* blooms. Limnol. Oceanogr. 50, 1020–1024. doi: 10.4319/lo.2005.50.3.1020

[ref55] Lewis J. D. Griffin K. L. Thomas R. B. Strain B. R. (1994). Phosphorus supply affects the photosynthetic capacity of loblolly pine grown in elevated carbon-dioxide. Tree Physiol. 14, 1229–1244. doi: 10.1093/treephys/14.11.1229, PMID: 14967614

[ref56] Li Z. K. Li W. Zhang Y. Hu Y. Y. Sheward R. Irwin A. J.. (2021). Dynamic photophysiological stress response of a model diatom to ten environmental stresses. J. Phycol. 57, 484–495. doi: 10.1111/jpy.13072, PMID: 32945529

[ref57] Li L. Lu S. H. Cen J. Y. (2019). Spatio-temporal variations of harmful algal blooms along the coast of Guangdong, southern China during 1980-2016. J. Oceanol. Limnol. 37, 535–551. doi: 10.1007/s00343-019-8088-y

[ref58] Li Y. Lu S. H. Jiang T. J. Xiao Y. P. You S. P. (2011). Environmental factors and seasonal dynamics of *Prorocentrum* populations in Nanji Islands National Nature Reserve East China Sea. Harmful Algae 10, 426–432. doi: 10.1016/j.hal.2010.08.002

[ref59] Li J. L. Sun X. X. (2016). Effects of different phosphorus concentrations and N/P ratios on the growth and photosynthetic characteristics of *Skeletonema costatum* and *Prorocentrum donghaiense*. Chin. J. Oceanol. Limn. 34, 1158–1172. doi: 10.1007/s00343-016-5169-z

[ref60] Lin Z. H. Chen L. S. Chen R. B. Zhang F. Z. Jiang H. X. Tang N. (2009). CO_2_ assimilation, ribulose-1,5-bisphosphate carboxylase/oxygenase, carbohydrates and photosynthetic electron transport probed by the JIP-test, of tea leaves in response to phosphorus supply. BMC Plant Biol. 9:43. doi: 10.1186/1471-2229-9-43, PMID: 19379526 PMC2685392

[ref61] Lin S. J. Litaker R. W. Sunda W. G. (2016). Phosphorus physiological ecology and molecular mechanisms in marine phytoplankton. J. Phycol. 52, 10–36. doi: 10.1111/jpy.12365, PMID: 26987085

[ref62] Liu Y. Li L. Zhai X. H. Zhou J. Ye P. H. Huang S. D. (2021). Analysis of the bloom caused by colonial *Phaeocystis globosa* in Mirs Bay. J. Trop. Oceanogr. 41, 164–171. doi: 10.11978/2021148

[ref63] Liu Y. Song X. X. Cao X. H. Yu Z. M. (2013). Responses of photosynthetic characters of *Skeletonema costatum* to different nutrient conditions. J. Plankton Res. 35, 165–176. doi: 10.1093/plankt/fbs080

[ref64] Liu C. L. Tang D. L. (2012). Spatial and temporal variations in algal blooms in the coastal waters of the western South China Sea. J. Hydro Environ. Res. 6, 239–247. doi: 10.1016/j.jher.2012.02.002

[ref66] Loebl M. Cockshutt A. M. Campbell D. A. Finkel Z. V. (2010). Physiological basis for high resistance to photoinhibition under nitrogen depletion in *Emiliania huxleyi*. Limnol. Oceanogr. 55, 2150–2160. doi: 10.4319/lo.2010.55.5.2150

[ref67] Lu S. H. Ou L. J. Dai X. F. Cui L. Dong Y. L. Wang P. B.. (2022). An overview of *Prorocentrum donghaiense* blooms in China: species identification, occurrences, ecological consequences, and factors regulating prevalence. Harmful Algae 114:102207. doi: 10.1016/j.hal.2022.102207, PMID: 35550289

[ref68] Ly J. Philippart C. J. M. Kromkamp J. C. (2014). Phosphorus limitation during a phytoplankton spring bloom in the western Dutch Wadden Sea. J. Sea Res. 88, 109–120. doi: 10.1016/j.seares.2013.12.010

[ref69] Malnoe A. Wang F. Girard-Bascou J. Wollman F. A. de Vitry C. (2014). Thylakoid FtsH protease contributes to photosystem II and cytochrome b(6)f remodeling in *Chlamydomonas reinhardtii* under stress conditions. Plant Cell 26, 373–390. doi: 10.1105/tpc.113.120113, PMID: 24449688 PMC3963582

[ref70] Markou G. Dao L. H. T. Muylaert K. Beardall J. (2017). Influence of different degrees of N limitation on photosystem II performance and heterogeneity of *Chlorella vulgaris*. Algal Res. 26, 84–92. doi: 10.1016/j.algal.2017.07.005

[ref71] Martin P. Van Mooy B. A. S. (2013). Fluorometric quantification of polyphosphate in environmental plankton samples: extraction protocols, matrix effects, and nucleic acid interference. Appl. Environ. Microb. 79, 273–281. doi: 10.1128/Aem.02592-12, PMID: 23104409 PMC3536087

[ref72] Meng X. Chen W. W. Wang Y. Y. Huang Z. R. Ye X. Chen L. S.. (2021). Effects of phosphorus deficiency on the absorption of mineral nutrients, photosynthetic system performance and antioxidant metabolism in *Citrus grandis*. PLoS One 16:e0246944. doi: 10.1371/journal.pone.0246944, PMID: 33596244 PMC7888624

[ref73] Moseley J. L. Chang C. W. Grossman A. R. (2006). Genome-based approaches to understanding phosphorus deprivation responses and PSR1 control in *Chlamydomonas reinhardtii*. Eukaryot. Cell 5, 26–44. doi: 10.1128/EC.5.1.26-44.2006, PMID: 16400166 PMC1360252

[ref74] Muller P. Li X. P. Niyogi K. K. (2001). Non-photochemical quenching. A response to excess light energy. Plant Physiol. 125, 1558–1566. doi: 10.1104/pp.125.4.1558, PMID: 11299337 PMC1539381

[ref75] Murata N. Nishiyama Y. (2018). ATP is a driving force in the repair of photosystem II during photoinhibition. Plant Cell Environ. 41, 285–299. doi: 10.1111/pce.13108, PMID: 29210214

[ref76] Neale P. J. Melis A. (1990). Activation of a reserve pool of photosystem II in *Chlamydomonas reinhardtii* counteracts photoinhibition. Plant Physiol. 92, 1196–1204. doi: 10.1104/pp.92.4.1196, PMID: 16667390 PMC1062435

[ref77] Nixon P. J. Michoux F. Yu J. F. Boehm M. Komenda J. (2010). Recent advances in understanding the assembly and repair of photosystem II. Ann. Bot. 106, 1–16. doi: 10.1093/aob/mcq059, PMID: 20338950 PMC2889791

[ref78] Oguz T. Merico A. (2006). Factors controlling the summer *Emiliania huxleyi* bloom in the Black Sea: a modeling study. J. Marine Syst. 59, 173–188. doi: 10.1016/j.jmarsys.2005.08.002

[ref79] Ou L. J. Huang X. Y. Huang B. Q. Qi Y. Z. Lu S. H. (2015). Growth and competition for different forms of organic phosphorus by the dinoflagellate *Prorocentrum donghaiense* with the dinoflagellate *Alexandrium catenella* and the diatom *Skeletonema costatum* s.l. Hydrobiologia 754, 29–41. doi: 10.1007/s10750-014-1994-2

[ref80] Ou L. J. Qin X. L. Shi X. Y. Feng Q. L. Zhang S. W. Lu S. H.. (2020). Alkaline phosphatase activities and regulation in three harmful *Prorocentrum* species from the coastal waters of the East China Sea. Microb. Ecol. 79, 459–471. doi: 10.1007/s00248-019-01399-3, PMID: 31267157

[ref81] Ou L. J. Wang D. Huang B. Q. Hong H. S. Qi Y. Z. Lu S. H. (2008). Comparative study of phosphorus strategies of three typical harmful algae in Chinese coastal waters. J. Plankton Res. 30, 1007–1017. doi: 10.1093/plankt/fbn058

[ref82] Pan X. L. Deng C. N. Zhang D. Y. Wang J. L. Mu G. J. Chen Y. (2008). Toxic effects of amoxicillin on the photosystem II of *Synechocystis* sp characterized by a variety of in vivo chlorophyll fluorescence tests. Aquat. Toxicol. 89, 207–213. doi: 10.1016/j.aquatox.2008.06.018, PMID: 18718680

[ref83] Pandey M. (2006). Nutrient modulated alkaline phosphatase and associated processes in diazotrophic cyanobacteria. Pol. J. Microbiol. 55, 53–62. 16878605

[ref84] Peperzak L. Colijn F. Gieskes W. W. C. Peeters J. C. H. (1998). Development of the diatom-*Phaeocystis* spring bloom in the Dutch coastal zone of the North Sea: the silicon depletion versus the daily irradiance threshold hypothesis. J. Plankton Res. 20, 517–537. doi: 10.1093/plankt/20.3.517

[ref85] Petrou K. Doblin M. A. Smith R. A. Ralph P. J. Shelly K. Beardall J. (2008). State transitions and non-photochemical quenching during a nutrient-induced fluorescence transient in phosphorus-starved *Dunaliella tertiolecta*. J. Phycol. 44, 1204–1211. doi: 10.1111/j.1529-8817.2008.00585.x, PMID: 27041717

[ref86] Popelkova L. Yocum C. F. (2011). PsbO, the manganese-stabilizing protein: analysis of the structure-function relations that provide insights into its role in photosystem II. J. Photochem. Photobiol. B 104, 179–190. doi: 10.1016/j.jphotobiol.2011.01.015, PMID: 21316983

[ref87] Reid P. C. Lancelot C. Gieskes W. W. C. Hagmeier E. Weichart G. (1990). Phytoplankton of the North Sea and its dynamics–a review. Neth. J. Sea Res. 26, 295–331. doi: 10.1016/0077-7579(90)90094-W

[ref88] Riegman R. Noordeloos A. A. M. Cadee G. C. (1992). Phaeocystis blooms and eutrophication of the continental coastal zones of the North Sea. Mar. Biol. 112, 479–484. doi: 10.1007/BF00356293

[ref89] Rosowski J. R. Parker B. C. (1971). Selected papers in phycology. Lincoln: Department of Botany, University of Nebraska.

[ref90] Schoemann V. Becquevort S. Stefels J. Rousseau W. Lancelot C. (2005). *Phaeocystis* blooms in the global ocean and their controlling mechanisms: a review. J. Sea Res. 53, 43–66. doi: 10.1016/j.seares.2004.01.008

[ref91] Schoemann V. Wollast R. Chou L. Lancelot C. (2001). Effects of photosynthesis on the accumulation of Mn and Fe by *Phaeocystis* colonies. Limnol. Oceanogr. 46, 1065–1076. doi: 10.4319/lo.2001.46.5.1065

[ref92] Semenov A. Y. Kurashov V. N. Mamedov M. D. (2011). Transmembrane charge transfer in photosynthetic reaction centers: some similarities and distinctions. J. Photochem. Photobiol. B. 104, 326–332. doi: 10.1016/j.jphotobiol.2011.02.004, PMID: 21356596

[ref93] Shelly K. Roberts S. Heraud P. Beardall J. (2005). Interactions between UV-B exposure and phosphorus nutrition. I. Effects on growth, phosphate uptake, and chlorophyll fluorescence. J. Phycol. 41, 1204–1211. doi: 10.1111/j.1529-8817.2005.00148.x

[ref95] Shi X. G. Lin X. Li L. Li M. Z. Palenik B. Lin S. J. (2017). Transcriptomic and microRNAomic profiling reveals multi-faceted mechanisms to cope with phosphate stress in a dinoflagellate. ISME J. 11, 2209–2218. doi: 10.1038/ismej.2017.81, PMID: 28548660 PMC5607363

[ref96] Solovchenko A. E. Ismagulova T. T. Lukyanov A. A. Vasilieva S. G. Konyukhov I. V. Pogosyan S. I.. (2019). Luxury phosphorus uptake in microalgae. J. Appl. Phycol. 31, 2755–2770. doi: 10.1007/s10811-019-01831-8

[ref97] Srivastava A. Guisse B. Greppin H. Strasser R. J. (1997). Regulation of antenna structure and electron transport in photosystem II of *Pisum sativum* under elevated temperature probed by the fast polyphasic chlorophyll a fluorescence transient: OKJIP. BBA Bioenergetics 1320, 95–106. doi: 10.1016/S0005-2728(97)00017-0

[ref98] Stirbet A. Govindjee N. R. (2012). Chlorophyll a fluorescence induction: a personal perspective of the thermal phase, the J-I-P rise. Photosynth. Res. 113, 15–61. doi: 10.1007/s11120-012-9754-5, PMID: 22810945

[ref99] Strasser R. J. Tsimilli-Michael M. Srivastava A. (2004). Analysis of the chlorophyll a fluorescence transient: Chlorophyll a fluorescence. Sign. Photosyn. 19, 321–362. doi: 10.1007/978-1-4020-3218-9_12

[ref100] Su W. W. Jakob T. Wilhelm C. (2012). The impact of nonphotochemical quenching of fluorescence on the photon balance in diatoms under dynamic light conditions. J. Phycol. 48, 336–346. doi: 10.1111/j.1529-8817.2012.01128.x, PMID: 27009723

[ref101] Thangaraj S. Palanisamy S. K. Zhang G. C. Sun J. (2021). Quantitative proteomic profiling of marine diatom *Skeletonema dohrnii* in response to temperature and silicate induced environmental stress. Front. Microbiol. 11:4832. doi: 10.3389/fmicb.2020.554832, PMID: 33519723 PMC7841394

[ref102] Thingstad T. F. Krom M. D. Mantoura R. F. C. Flaten G. A. F. Groom S. Herut B.. (2005). Nature of phosphorus limitation in the ultraoligotrophic eastern Mediterranean. Science 309, 1068–1071. doi: 10.1126/science.1112632, PMID: 16099984

[ref103] Tyrrell T. Merico A. (2004). *Emiliania huxleyi*: bloom observations and the conditions that induce them. Coccolithophores: from molecular processes to global impact eds. Thierstein H. R. Young J. R. (Berlin, Heidelberg: Springer), 75–97.

[ref104] Van Rensen J. J. S. Vredenberg W. J. (2009). Higher concentration of Q(B)-nonreducing photosystem II centers in triazine-resistant *Chenopodium album* plants as revealed by analysis of chlorophyll fluorescence kinetics. J. Plant Physiol. 166, 1616–1623. doi: 10.1016/j.jplph.2009.04.011, PMID: 19477550

[ref105] Vass I. Kirilovsky D. Etienne A. L. (1999). UV-B radiation-induced donor-side and acceptor-side modifications of photosystem II in the cyanobacterium *Synechocystis* sp PCC 6803. Biochemistry 38, 12786–12794. doi: 10.1021/bi991094w, PMID: 10504248

[ref106] Veldhuis M. J. W. Colijn F. Admiraal W. (1991). Phosphate utilization in *Phaeocystis pouchetii* (Haptophyceae). Pszni. Mar. Ecol. 12, 53–62. doi: 10.1111/j.1439-0485.1991.tb00083.x

[ref107] Verity P. G. Medlin L. K. (2003). Observations on colony formation by the cosmopolitan phytoplankton genus *Phaeocystis*. J. Mar. Syst. 43, 153–164. doi: 10.1016/j.jmarsys.2003.09.001

[ref108] Veronica N. Subrahmanyam D. Kiran T. V. Yugandhar P. Bhadana V. P. Padma V.. (2017). Influence of low phosphorus concentration on leaf photosynthetic characteristics and antioxidant response of rice genotypes. Photosynthetica 55, 285–293. doi: 10.1007/s11099-016-0640-4

[ref109] Wang K. Chen B. H. Gao Y. H. Lin H. (2021). Harmful algal blooms caused by *Phaeocystis globosa* from 1997 to 2018 in Chinese coastal waters. Mar. Pollut. Bull. 173:112949. doi: 10.1016/j.marpolbul.2021.112949 34547638

[ref110] Wang Y. Deng K. Wang X. D. (2013). The effects of light, nutrient and co-existing diatom on colony formation of *Phaeocystis globosa*. Ecol. Sci. 32, 165–170.

[ref111] Wang X. D. Huo Y. P. Yang F. Wang Y. (2021). Induced Allelopathic effects of *Thalassiosira weissflogii* on Colony formation in *Phaeocystis globosa*. Water-Sui 13:581. doi: 10.3390/w13050581

[ref112] Wang Z. H. Liu L. Tang Y. L. Li A. Liu C. Xie C.. (2022). Phytoplankton community and HAB species in the South China Sea detected by morphological and metabarcoding approaches. Harmful Algae 118:102297. doi: 10.1016/j.hal.2022.102297, PMID: 36195422

[ref113] White A. Dyhrman S. (2013). The marine phosphorus cycle. Front. Microbiol. 4:105. doi: 10.3389/fmicb.2013.00105, PMID: 23734145 PMC3659303

[ref114] Wilson A. J. Jain P. K. (2018). Structural dynamics of the oxygen-evolving complex of photosystem II in water-splitting action. J. Am. Chem. Soc. 140, 5853–5859. doi: 10.1021/jacs.8b02620, PMID: 29649874

[ref115] Wu H. Y. Cockshutt A. M. McCarthy A. Campbell D. A. (2011). Distinctive photosystem II photoinactivation and protein dynamics in marine diatoms. Plant Physiol. 156, 2184–2195. doi: 10.1104/pp.111.178772, PMID: 21617029 PMC3149953

[ref116] Wu H. Roy S. Alami M. Green B. R. Campbell D. A. (2012). Photosystem II photoinactivation, repair, and protection in marine centric diatoms (vol 160, pg 464, 2012). Plant Physiol. 160:1146. doi: 10.1104/pp.112.900447 PMC3440219 22829321

[ref117] Wykoff D. D. Davies J. P. Melis A. Grossman A. R. (1998). The regulation of photosynthetic electron transport during nutrient deprivation in *Chlamydomonas reinhardtii*. Plant Physiol. 117, 129–139. doi: 10.1104/pp.117.1.129, PMID: 9576782 PMC34996

[ref118] Yang Y. J. Shi J. Q. Jia Y. L. Bai F. Yang S. Q. Mi W. M.. (2020). Unveiling the impact of glycerol phosphate (DOP) in the dinoflagellate *Peridinium bipes* by physiological and transcriptomic analysis. Environ. Sci. Eur. 32:38. doi: 10.1186/s12302-020-00317-6

[ref119] Yu Z. B. Lu Y. Du J. J. Peng J. J. Wang X. Y. (2014). The chloroplast protein LTO1/AtVKOR is involved in the xanthophyll cycle and the acceleration of D1 protein degradation. J. Photochem. Photobiol. B. 130, 68–75. doi: 10.1016/j.jphotobiol.2013.11.003, PMID: 24300993

[ref121] Zhang X. Ma F. Zhu X. Zhu J. Y. Rong J. F. Zhan J.. (2017). The acceptor side of photosystem II is the initial target of nitrite stress in *Synechocystis* sp Strain PCC 6803. Appl. Environ. Microbiol. 83:16. doi: 10.1128/AEM.02952-16, PMID: 27864175 PMC5244309

[ref122] Zhang S. F. Yuan C. J. Chen Y. Chen X. H. Li D. X. Liu J. L.. (2016). Comparative transcriptomic analysis reveals novel insights into the adaptive response of *Skeletonema costatum* to changing ambient phosphorus. Front. Microbiol. 7:1476. doi: 10.3389/fmicb.2016.01476, PMID: 27703451 PMC5028394

[ref123] Zhao Y. Wang Y. Quigg A. (2015). Comparison of population growth and photosynthetic apparatus changes in response to different nutrient status in a diatom and a coccolithophore. J. Phycol. 51, 872–884. doi: 10.1111/jpy.12327, PMID: 26986884

[ref125] Zhu X. G. Govindjee Baker N. R. deSturler E. Ort D. R. Long S. P. (2005). Chlorophyll a fluorescence induction kinetics in leaves predicted from a model describing each discrete step of excitation energy and electron transfer associated with photosystem II. Planta 223, 114–133. doi: 10.1007/s00425-005-0064-4, PMID: 16411287

[ref126] Zhuang J. L. Lu J. C. Cao K. F. Li J. (2022). Haploid helps *Phaeocystis globosa* distribute to deeper dim water, as evidenced by growth and photosynthetic physiology. Front. Mar. Sci. 9:2330. doi: 10.3389/fmars.2022.902330

