# Supplementary figures and images for: Comparison of photosynthetic responses between haptophyte Phaeocystis globosa and diatom Skeletonema costatum under phosphorus limitation
Source: Front Microbiol. 2023 Jan 23;14:1085176. doi: 10.3389/fmicb.2023.1085176 (PMC9899818; doi:10.3389/fmicb.2023.1085176)

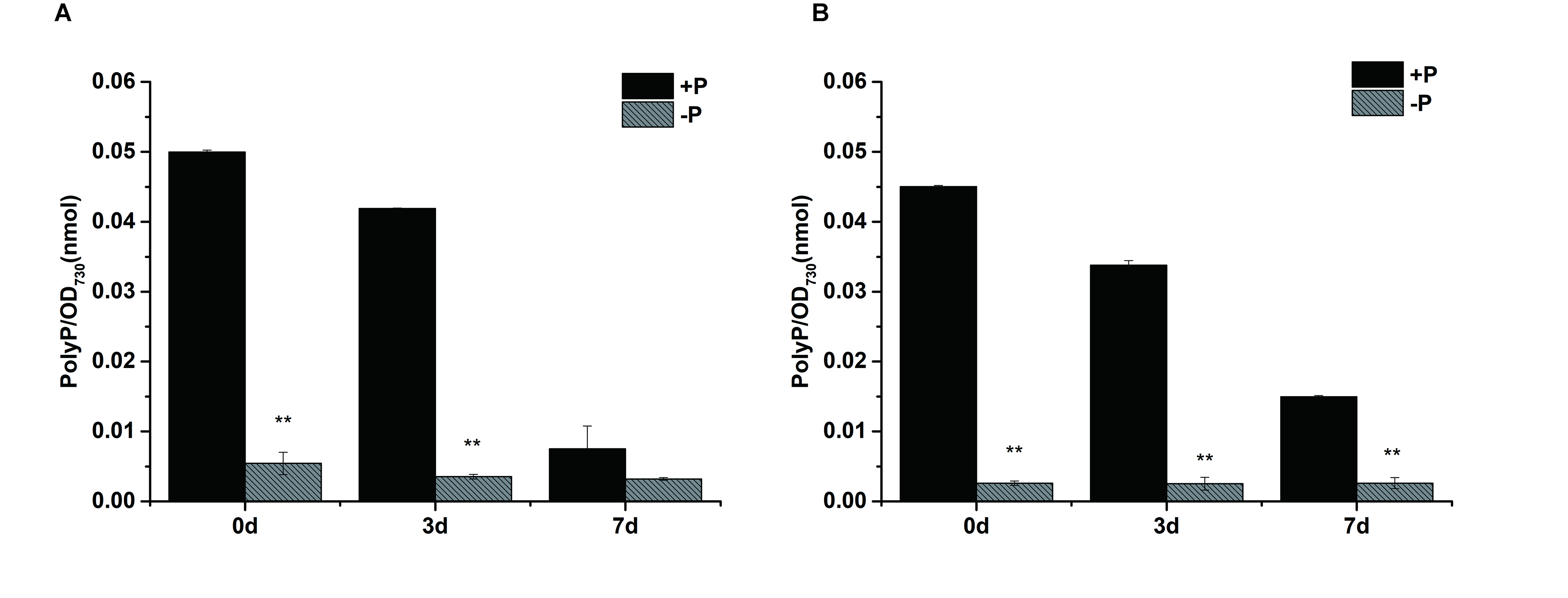

Supplement: Supplementary file 1 [file Image_1.TIF]

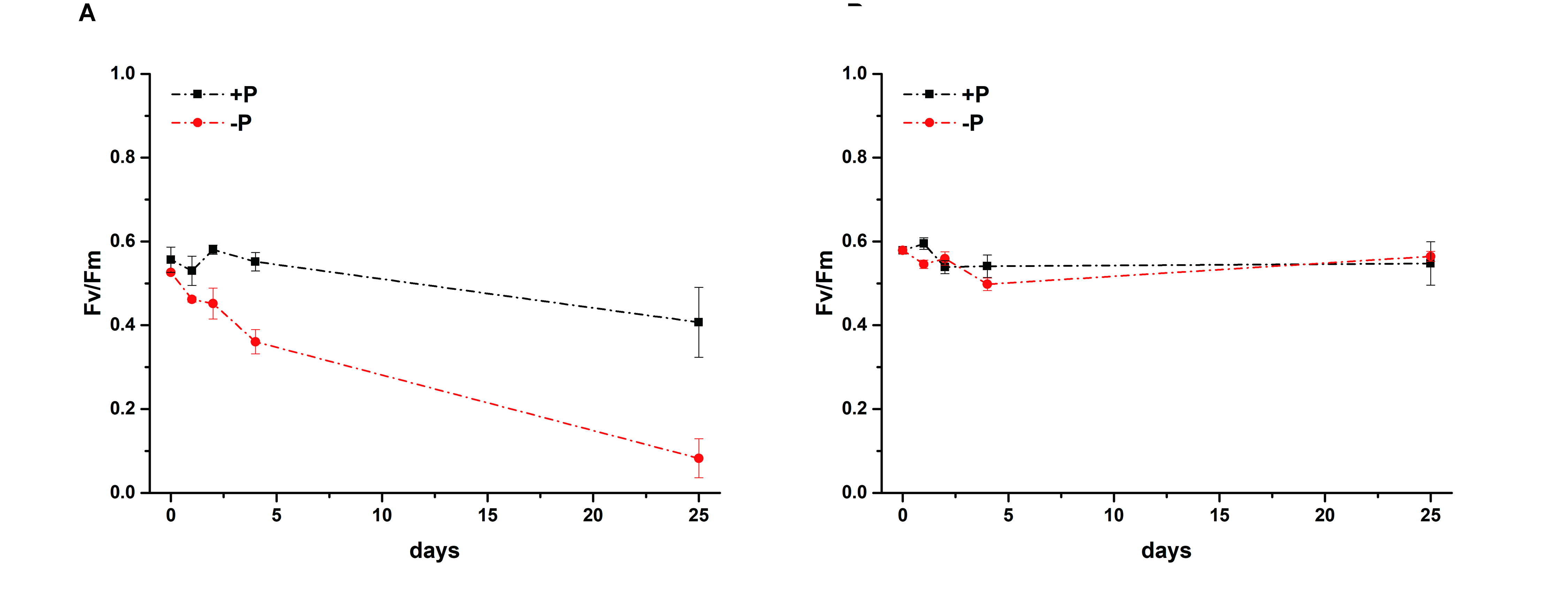

Supplement: Supplementary file 2 [file Image_2.TIF]

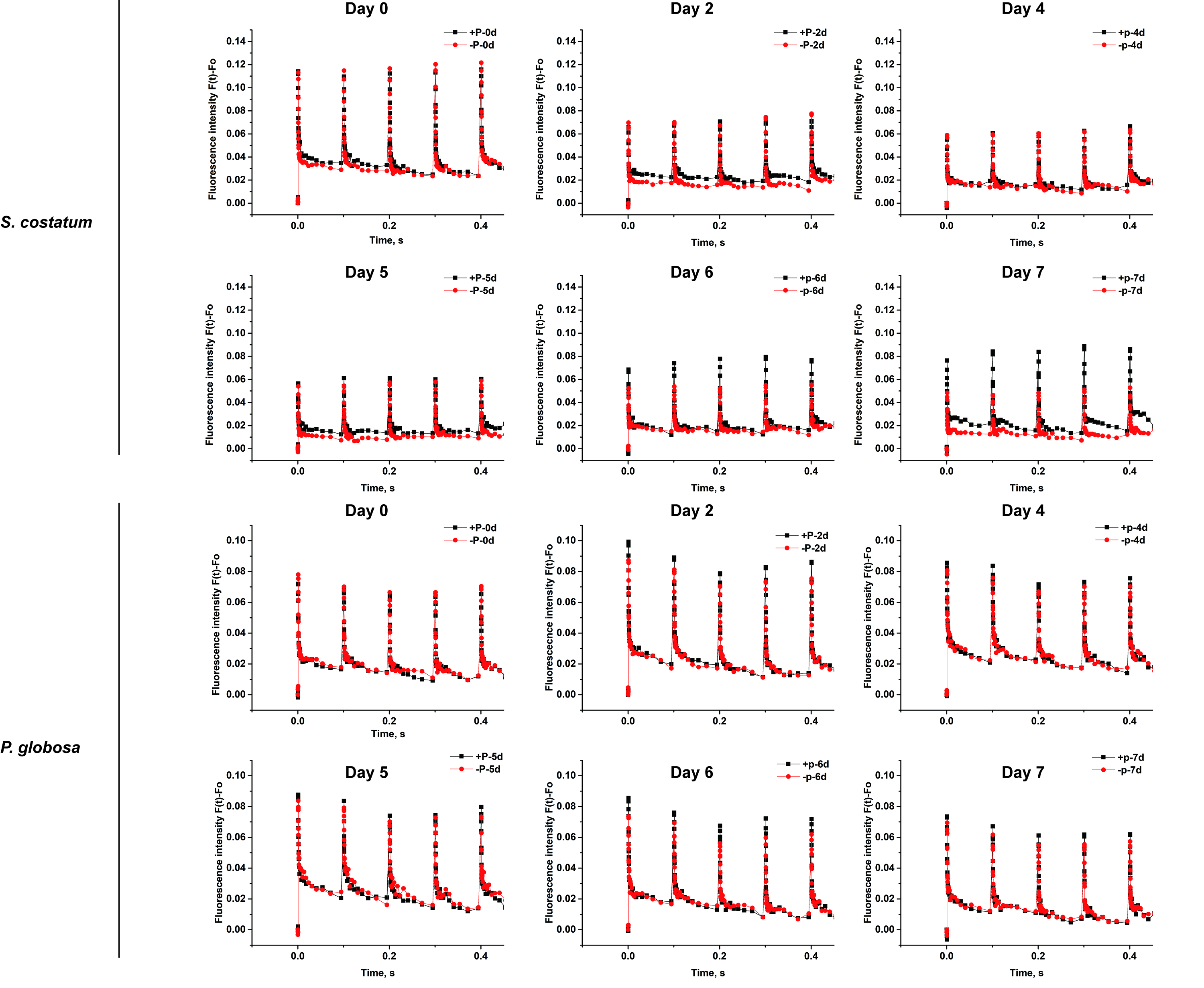

Supplement: Supplementary file 3 [file Image_3.TIF]

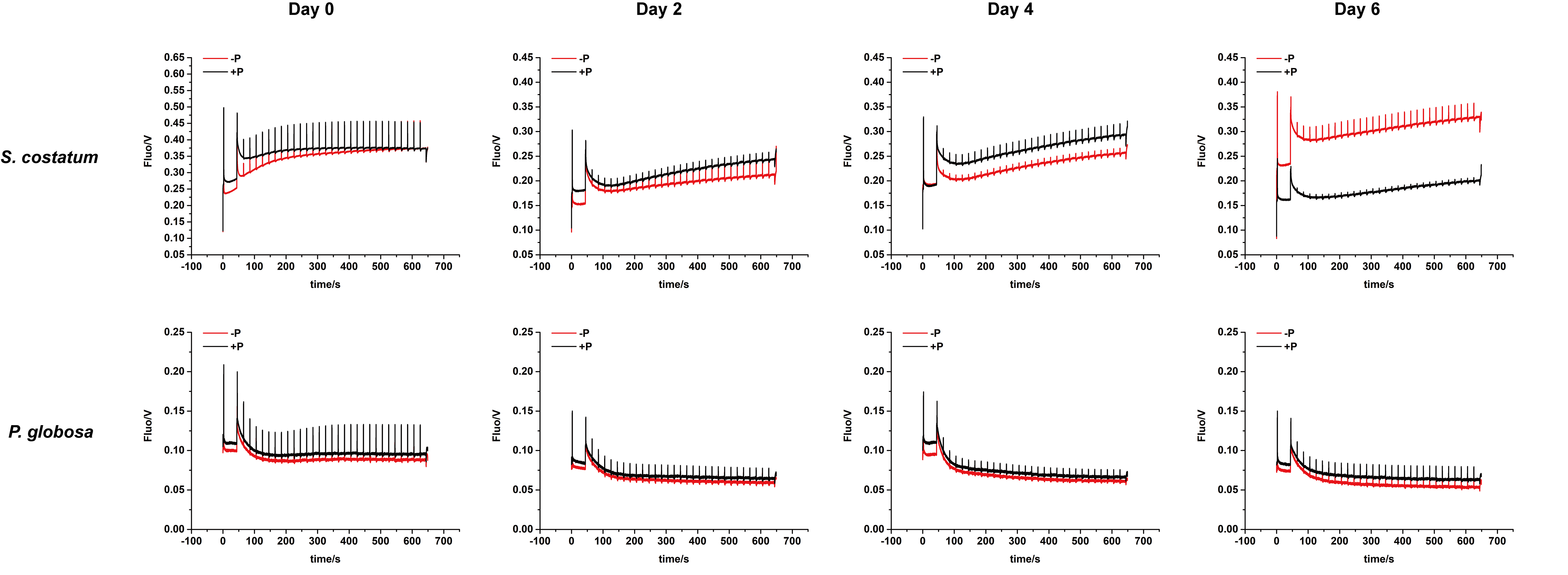

Supplement: Supplementary file 4 [file Image_4.TIF]
