# Supplementary material for: Comparison of photosynthetic responses between haptophyte Phaeocystis globosa and diatom Skeletonema costatum under phosphorus limitation
Source: Front Microbiol. 2023 Jan 23;14:1085176. doi: 10.3389/fmicb.2023.1085176 (PMC9899818; doi:10.3389/fmicb.2023.1085176)
Supplement: Supplementary file 5 [file Table_1.docx]

**Supplementary Table**

**Supplementary Table 1**. The recipe of Erdschreiber's medium

| Component | Amount | Stock solution concentration | Final concentration |
| --- | --- | --- | --- |
| Pasteurized seawater | 3 L |  |  |
| P−IV metal solution^*^ | 36 mL/3 L |  |  |
| NaNO_3_ (autoclave before adding) | 10 mL/3 L | 0.7 M | 2.3 mM |
| Na_2_HPO_4_·7H_2_O (autoclave before adding) | 10 mL/3 L | 0.02 M | 0.067 mM |
| Soilwater | 150 mL/3 L |  |  |
| Vitamin B_12_ | 3 mL/3 L | 0.027 g/200 mL |  |

**^*^: P−IV metal solution**

| Component | Amount | Final concentration |
| --- | --- | --- |
| Na_2_EDTA·2H_2_O | 0.75 g/L | 2 mM |
| FeCl_3_·6H_2_O | 0.097 g/L | 0.36 mM |
| MnCl_2_·4H_2_O | 0.041 g/L | 0.21 mM |
| ZnCl_2_ | 0.005 g/L | 0.037 mM |
| CoCl_2_·6H_2_O | 0.002 g/L | 0.0084 mM |
| Na_2_MoO_4_·2H_2_O | 0.004 g/L | 0.017 mM |

**Supplementary figure caption**

**Supplementary Figure S1**. The polyphosphate body (PolyP) content in *S. costatum* and *P. globosa* in +P and −P groups on days 0, 3 and 7.

**Supplementary Figure S2**. Maximum photochemistry efficiency (F_v_/F_m_) values of *S. costatum* (A) and *P. globosa* (B) cultured for 25 days in +P and −P groups.

**Supplementary Figure S3**. Fluorescence decay curves induced in *S. costatum* (A) and *P. globosa* (B) in +P and −P groups for various periods of time.

**Supplementary Figure S4**. The slow induction kinetic curves of *S. costatum* and *P. globosa* in +P and −P groups for various periods of time.
